# Rapid detection of enterotoxigenic *Bacillus cereus* by loop-mediated isothermal amplification assay from food and feed samples

**DOI:** 10.3389/fcimb.2026.1815090

**Published:** 2026-06-19

**Authors:** Md Atiqul Haque, Md Arifur Rahman, Israt Islam, Uditi Paul Bristi, Farzana Islam, Nazmul Alam, Tanjina Sultana, Jubayer Mumin, Firoz Ahmed, Cheng He, Md Aminul Islam

**Affiliations:** 1 Department of Microbiology, Faculty of Veterinary and Animal Science, Hajee Mohammad Danesh Science and Technology University, Dinajpur, Bangladesh; 2 Department of Microbiology, Faculty of Science, Noakhali Science and Technology University, Noakhali, Bangladesh; 3 Department of Obstetrics and Gynecology, 250 Bedded General Hospital, Noakhali, Bangladesh; 4 Department of Global Public Health, Karolinska Institute, Stockholm, Sweden; 5 National Key Laboratory of Veterinary Public Health, College of Veterinary Medicine, China Agricultural University, Beijing, China; 6 Advanced Molecular Lab, Department of Microbiology, President Abdul Hamid Medical College, Kishoreganj, Bangladesh

**Keywords:** *Bacillus cereus*, Bangladesh, *cytK*, enterotoxin, *entFM*, food safety, loop-mediated isothermal amplification (LAMP), *nheA*

## Abstract

**Background:**

Foodborne illnesses caused by enterotoxigenic *Bacillus cereus* represent a significant public health concern globally. Rapid and simple detection methods are needed, particularly in resource-limited settings where conventional laboratory infrastructure is lacking. This study aimed to develop and validate a loop-mediated isothermal amplification (LAMP) assay targeting three major enterotoxin genes (*nheA*, *cytK*, and *entFM*) for the rapid detection of enterotoxigenic *B*. *cereus* in food and feed samples from Bangladesh.

**Methods:**

LAMP primers for the *nheA* gene were newly designed, while *cytK* and *entFM* primers were adapted from previous studies. Reaction conditions were optimized using *B*. *cereus* ATCC 14579. Analytical sensitivity was determined using serial dilutions of pure culture, spiked milk, and genomic DNA. Specificity was assessed against 20 bacterial strains, including *Bacillus* species and non-*Bacillus* foodborne pathogens. The assay was validated on 30 field samples (feed, milk, and eggs) and compared with conventional PCR.

**Results:**

Optimal LAMP amplification was achieved at 64°C for 40 min. The limit of detection was 100 fg/µL for genomic DNA, 9 × 10^2^ CFU/mL for pure culture, and 9 × 10^3^ CFU/mL for spiked milk—approximately 10,000-fold more sensitive than conventional PCR. The assay showed 100% specificity for enterotoxigenic *Bacillus* species, with no cross-amplification of non-target bacteria. In field samples, LAMP detected enterotoxin genes in 73.3% (22/30) for *nheA*, 53.3% (16/30) for *cytK*, and 80.0% (24/30) for *entFM*, compared to 63.3% (19/30), 40.0% (12/30), and 70.0% (21/30) by PCR, respectively. Overall diagnostic sensitivity and specificity against PCR were 96.1% and 66.7%, respectively, with substantial agreement (κ = 0.62).

**Conclusion:**

Although LAMP assays for individual *B*. *cereus* toxin genes have been described previously, this study represents the first application of this specific *nheA*-*cytK*-*entFM* LAMP panel in the Bangladeshi food and feed context, providing a regionally relevant, field-deployable method for food safety monitoring.

## 1 Introduction

*Bacillus cereus* (*B*. *cereus*) is a Gram-positive, rod-shaped spore-forming bacterium widely distributed in nature and commonly associated with foodborne illnesses (Haque et al., 2021a; Wang et al., 2022). This opportunistic pathogen contaminates various foods and animal feeds, causing significant economic losses in the livestock industry and posing serious health risks to consumers (Haque et al., 2021b; Wang et al., 2022; Yun-Ming et al., 2022). *B*. *cereus* food poisoning manifests as either diarrheal or emetic syndromes, with the diarrheal form being more frequently associated with animal-derived foods (Dietrich et al., 2021). In immunocompromised individuals, the elderly, and neonates, *B*. *cereus* can also cause systemic infections, including bacteremia, endocarditis, and meningitis (Haque et al., 2022).

The pathogenicity of *B*. *cereus* is attributed to multiple virulence factors, particularly enterotoxins. The non-hemolytic enterotoxin (NHE) complex, encoded by the *nheABC* genes, is considered the primary diarrheal toxin, present in 85%–100% of clinical isolates (Dietrich et al., 2021; Haque et al., 2021b). Cytotoxin K (CytK), a potent pore-forming toxin, and enterotoxin FM (EntFM) are additional major virulence factors contributing to diarrheal disease (Li et al., 2020; Zuo et al., 2020). Our previous studies in Bangladesh revealed high prevalence of these enterotoxin genes among *B*. *cereus* isolates from food and feed samples, with *nheA* detected in >80%, *entFM* in 80%, and *cytK* in 71% of isolates (Haque et al., 2022). This high prevalence underscores the need for effective surveillance methods in the region. The *nheA*, *cytK*, and *entFM* genes are potential enterotoxigenic *Bacillus cereus*-group-specific targets, as they are present in nearly all enteropathogenic *Bacillus* species and are considered major diarrhea-causing virulence factors (Ceuppens et al., 2013). It is essential to design a quick, sensitive, and easy technique for identifying *B*. *cereus* to improve food safety and safeguard public health.

Conventional culture-based methods for *B*. *cereus* detection, while reliable, are time-consuming (3–5 days), labor-intensive, and require specialized laboratory facilities (Liu et al., 2011). Immunological techniques such as ELISA and reverse passive latex agglutination offer faster results but suffer from limited sensitivity and cross-reactivity issues (Bao et al., 2020). Polymerase chain reaction (PCR)-based methods, including conventional, real-time, and multiplex PCR, provide improved sensitivity and specificity but require expensive thermal cyclers and trained personnel, limiting their applicability in resource-limited settings (Deng et al., 2020). Furthermore, PCR is susceptible to inhibition by food matrix components, potentially yielding false-negative results (Mandappa et al., 2016).

Loop-mediated isothermal amplification (LAMP), developed by Notomi et al. (2000), overcomes many limitations of conventional nucleic acid amplification methods. LAMP amplifies DNA under isothermal conditions (60°C–65°C) using a set of 4–6 primers recognizing 6–8 regions of the target gene, achieving high specificity and sensitivity (10^9^-fold amplification in <60 min). The method requires only a simple heating device (water bath or heating block), and results can be visualized by color change or turbidity, making it ideal for field deployment and resource-limited laboratories (Notomi et al., 2000; Yun-Ming et al., 2022). Previous LAMP assays for *B*. *cereus* have targeted individual enterotoxin genes, including *nheB*, *hblA*, *entFM*, and the emetic toxin gene *ces* (Liu et al., 2011; Mandappa et al., 2016; Busch et al., 2022; Yun-Ming et al., 2022). These three genes were selected because: (i) they encode the major diarrheal enterotoxins responsible for food poisoning; (ii) they show high prevalence among Bangladeshi *B*. *cereus* isolates in our previous study; (iii) their combined detection provides comprehensive coverage of diarrheal-type virulence potential; and (iv) the emetic toxin gene *ces* was not included, as our focus was on diarrheal-type food poisoning, which is more commonly associated with animal-derived foods.

However, to our knowledge, no validated LAMP method targeting this specific combination of enterotoxin genes has been reported for food and feed samples tested within Bangladesh, where *B*. *cereus* contamination poses significant food safety risks. A LAMP assay targeting multiple enterotoxin genes can provide rapid, sensitive, and specific detection of enterotoxigenic *B*. *cereus* directly from food and feed samples without culture-based pre-enrichment, with performance superior to conventional PCR and suitability for field deployment in resource-limited settings. This study aimed to develop and validate a LAMP-based method for detecting enterotoxigenic *B*. *cereus* in food and feed samples from Bangladesh, targeting three major enterotoxin genes, and to evaluate its diagnostic performance against conventional PCR.

## 2 Methods

### 2.1 Bacterial strains, culture conditions, and DNA extraction

A total of 20 bacterial strains were used in this study (**Table 1**). Six *Bacillus* strains included *B*. *cereus* ATCC 14579 (reference strain), *B*. *cereus* OP659027.1, *B*. *cereus* OP672318.1, *B*. *thuringiensis* OP659032.1, *Bacillus* spp. OP659031.1, and *Bacillus* spp. OP660549.1 (previously isolated from Bangladeshi food samples; Haque et al., 2022). Eight non-*Bacillus* strains comprised *Staphylococcus aureus* ATCC 25923, *Shigella flexneri* ATCC 12022, *Klebsiella pneumoniae* ATCC 700603, *Escherichia coli* ATCC 25922, *Enterococcus faecalis* ATCC 29212, *Salmonella* Typhimurium ATCC 14028, *Listeria monocytogenes* ATCC 19115, and *Campylobacter jejuni* ATCC 33560. For expanded specificity testing, additional *Bacillus* species (*B*. *mycoides* ATCC 6462, *B*. *cytotoxicus* ATCC 10987, *B*. *weihenstephanensis* DSM 11821, *B*. *anthracis* ATCC 4229, *B*. *subtilis* ATCC 6633, and *B*. *licheniformis* ATCC 14580) were obtained from culture collections.

**Table 1:** The bacterial strains used in this study.

S/N	Strain ID	Bacterial species	Source	Results	
				LAMP	PCR
1	ATCC 14579	*Bacillus cereus*	ATCC	+	+
2	OP659027.1	*B*. *cereus*	Bangladesh isolate	+	+
3	OP672318.1	*B. cereus*	Bangladesh isolate	+	+
4	OP659032.1	*B*. *thuringiensis*	Bangladesh isolate	+	+
5	OP659031.1	*Bacillus* spp.	Bangladesh isolate	+	+
6	OP660549.1	*Bacillus* spp.	Bangladesh isolate	+	+
7	ATCC 10987	*B. cytotoxicus*	ATCC	+	+
8	ATCC 6462	*B. mycoides*	ATCC	–	–
9	DSM 11821	*B. weihenstephanensis*	DSM	–	–
10	ATCC 4229	*B. anthracis*	ATCC	–	–
11	ATCC 6633	*B. subtilis*	ATCC	–	–
12	ATCC 14580	*B. licheniformis*	ATCC	–	–
13	ATCC 25923	*Staphylococcus aureus*	ATCC	–	–
14	ATCC 12022	*Shigella flexneri*	ATCC	–	–
15	ATCC 700603	*Klebsiella pneumoniae*	ATCC	–	–
16	ATCC 25922	*Escherichia coli*	ATCC	–	–
17	ATCC 29212	*Enterococcus faecalis*	ATCC	–	–
18	ATCC 14028	*Salmonella* Typhimurium	ATCC	–	–
19	ATCC 19115	*Listeria monocytogenes*	ATCC	–	–
20	ATCC 33560	*Campylobacter jejuni*	ATCC	–	–

All strains were stored at -80°C in Tryptic Soy Broth (TSB) containing 25% glycerol. Working cultures were prepared by streaking onto appropriate selective media: MYP agar (HiMedia, India) for *Bacillus* spp., MS agar (HiMedia, India) for *S*. *aureus*, XLD agar (HiMedia, India) for *Shigella* and *Salmonella*, EMB agar (HiMedia, India) for *E*. *coli*, ES agar (Sigma-Aldrich, Germany) for *E*. *faecalis*, and PALCAM agar (HiMedia, India) for *Listeria*. Plates were incubated at 37°C for 18–24 h. Single colonies were subcultured in LB broth (HiMedia, India) at 37°C with shaking (120 rpm) for DNA extraction (Microbiology Laboratory Guidebook [MLG], 2015; Akter et al., 2020; Zaman Faruk et al., 2025).

Genomic DNA from pure bacterial cultures was extracted using the TaKaRa MiniBEST Bacteria Genomic DNA Extraction Kit Ver. 3.0 (GW Vitek, Seoul, Korea, Cat. #9763) according to the manufacturer’s instructions. DNA concentration and purity were measured using a NanoDrop™ 8000 spectrophotometer (Thermo Scientific, USA). DNA samples with A260/A280 ratios between 1.8–2.0 were used for experiments. Extracted DNA was stored at -20°C until use.

For rapid processing of field samples without culture-based pre-enrichment, a simple boiling method was adapted from Sowmya et al. (2012) with modifications. Briefly, 1 g of feed samples or 1 mL of liquid samples was suspended in 2 mL of sterile PBS. The suspensions were first centrifuged at 2,000 × g for 2 min at 4°C to remove debris. The resulting supernatants were transferred to fresh tubes and centrifuged at 8,000 × g for 10 min at 4°C. The pellets were then resuspended in 100 μL of 1% Triton X-100 (Sigma-Aldrich, Germany, Cat# T8787). These suspensions were boiled at 100°C for 10 min in a heating block. After boiling, the lysates were centrifuged at 12,000 × g for 5 min at 4°C. Finally, 5 μL of supernatant was used directly for LAMP or stored at -20°C.

The boiling method was validated against kit extraction using 10 representative field samples (two from each of five matrices: poultry feed, cattle feed, fish feed, milk, and egg). DNA yield, purity, and amplification success rates were compared (**Supplementary Table 1**), which demonstrated that the simple boiling method yields DNA of sufficient quantity and quality for LAMP amplification in most samples, supporting our conclusion that complex purification is not required for field applications. PCR inhibition was assessed by spiking DNA extracts with 1 ng of positive control DNA and observing amplification success.

All LAMP and PCR reactions included an internal amplification control (IAC) targeting the *B*. *cereus 16S rRNA* gene (primers: F-5’-GCGGCGTGCCTAATACATGC-3’, R-5’-CTCAGGTCGGCTACGCATCG-3’) (Zhang et al., 2014) to monitor for PCR inhibition. Samples showing IAC failure were re-extracted and retested after a 1:5 dilution.

### 2.2 Target gene selection, primer design, and LAMP reaction optimization

Three enterotoxin genes were targeted: *nheA* (encoding non-hemolytic enterotoxin A), *cytK* (encoding cytotoxin K), and *entFM* (encoding enterotoxin FM). These genes were selected based on: (i) their role as major diarrheal virulence factors; (ii) high prevalence (>70%) in Bangladeshi *B*. *cereus* isolates (Haque et al., 2022); and (iii) suitability for LAMP primer design.

Novel LAMP primers targeting the *nheA* gene (GenBank accession AJI04953.1) were designed using Primer Explorer V4 software (http://primerexplorer.jp/elamp4.0.0/). Primer sets (F3, B3, FIP, BIP, LF, and LB) (**Table 2**) were selected based on standard LAMP design criteria. LAMP primers for *cytK* (DQ019311.1) and *entFM* (AY789084.1) (**Table 2**) were adapted from Mandappa et al. (2016) with sequence verification against Bangladeshi isolates.

**Table 2:** LAMP primers used in this study.

Target gene	Primer	Sequence (5’→3’)	Length (bp)	Tm (°C)	GC%	Reference
*nheA* (AJI04953.1)^1^	nheA-F3	GGCAAACAGAAGTGAAAACAG	21	60.2	45	This study
	nheA-B3	TTAAGTCAATTAGCTTCGGATT	22	59.8	36	
	nheA-FIP	GGTGACTGTGATCCTAACATTCTAAGCACAAAATGTAATTGCTCCAA	47	64.1	40	
	nheA-BIP	CAACAGCCAGACATTAAGGTAAATGACTCTCTTACATTTGCCTTTG	46	63.8	41	
	nheA-LF	GGTGACTGTGATCCTAACATTCTA	24	61.2	42	
	nheA-LB	CGATGAGTAGTTTGACGAATCATCA	25	60.9	40	
*cytK* (DQ019311.1)^1^	cytK-F3	CTAGCGTATCTTATCAACTTGG	22	59.7	45	Mandappa et al., 2016
	cytK-B3	CCGTTAAAGAATACGTTCCAT	21	58.9	43	
	cytK-FIP	GACCAGTTGCACCAGCTTCATGGCTCTGTTAAAGCTTCTG	40	64.5	53	
	cytK-BIP	AAGTCACTTGGTCTGACTCTGTTTTACGTTTTTGTTCGTTTGG	43	63.9	42	
	cytK-LF	GACCAGTTGCACCAGCTTCAT	21	62.1	57	
	cytK-LB	CGCTAGGGCCATTAGGCGT	19	61.8	68	
*entFM* (AY789084.1)^1^	entFM-F3	GGTTATGTAAGTGCAGACTTC	21	59.5	48	Mandappa et al., 2016
	entFM-B3	TCAAAACCAGCAGGTGTT	18	58.2	44	
	entFM-FIP	CGTCTTTACCTGGTTGTTGAACGGTTAAGTTTGTAAAAGGCGGA	44	64.2	45	
	entFM-BIP	CCAACAACAGGTGGAGATACATCGCAGTTCTGTATGGTGAAC	42	64.0	48	
	entFM-LF	TCGCTGGATTCGCTAGATCTTT	22	61.3	45	
	entFM-LB	GGTATTGCTGATGGAAACACAG	22	60.8	45	

1 GenBank accession number; F3, Forward outer primer; B3, Backward outer primer; FIP Forward inner primer; BIP, Backward inner primer; LF, Loop forward primer; LB, Loop backward primer.

All LAMP primers were analyzed using BLASTn against the NCBI non-redundant nucleotide database (accessed March 2024). The *nheA* primers showed 100% identity with 47 *B*. *cereus* and 43 *B*. *thuringiensis* sequences. For cytK and entFM primers, sequence validation against 15 local Bangladeshi *B*. *cereus* isolates showed 100% homology across all isolates with no mismatches. No significant homology (>70% coverage, >80% identity) was observed with non-*Bacillus* foodborne pathogens, including *Salmonella*, *Listeria*, *E*. *coli*, *Klebsiella*, *Shigella*, *Enterococcus*, *Campylobacter*, and *Staphylococcus* species. Primer secondary structures (hairpins, self-dimers, and cross-dimers) were evaluated using the OligoAnalyzer Tool (IDT). All primers showed ΔG values ranging from -0.7 to -2.5 kcal/mol for hairpin formation and -2.8 to -5.6 kcal/mol for dimer formation, indicating minimal risk of non-specific structures. Complete thermodynamic data are provided in **Supplementary Tables 2A–D**.

Conventional PCR primers for all three genes were as previously described (Haque et al., 2022) and are provided in **Supplementary Table 3**. All primers were synthesized by Macrogen, Inc. (Seoul, South Korea).

LAMP reactions were carried out in a total volume of 25 μL. The reaction mixture consisted of 12.5 μL WarmStart^®^ Colorimetric LAMP 2X Master Mix (New England Biolabs, Cat# M1800S), 1.6 μM each of the primers FIP and BIP (40 pmol each), 0.2 μM each of the outer primers F3 and B3 (5 pmol each), and 0.8 μM each of the loop primers LF and LB (20 pmol each). Subsequently, 5 μL of DNA template was added, which contained either 10–100 ng DNA from pure culture or 5 μL crude lysate from field samples. Finally, the volume was adjusted to 25 μL with nuclease-free water.

Reactions were incubated at 61°C, 62°C, 63°C, 64°C, and 65°C for 40 min in a Labnet D1301-230V AccuBlock™ Dry Block Heater (Cole-Parmer, USA), followed by 80°C for 2 min to terminate the reaction. At the optimal temperature, reactions were incubated for 10, 20, 30, and 40 min to determine the minimum time required for detectable amplification. Reactions were incubated in a heating block, and the tubes were visually inspected against a white background. Positive reactions showed a distinct color change from pink/orange to yellow. Color was recorded by two independent observers and scored as: ++ (bright yellow, clearly visible), + (pale yellow, visible but weak), ± (ambiguous), – (pink/orange). Products (5 μL) were electrophoresed on 1.5% agarose gels in 1× TAE buffer at 100 V for 40 min, stained with ethidium bromide (0.5 μg/mL), and visualized under UV light using a Gel Doc™ EZ System (Bio-Rad, USA). All ambiguous and negative results were confirmed by gel electrophoresis. For quantitative assessment, absorbance at 420 nm was measured for a subset of samples using a spectrophotometer (Eppendorf BioSpectrometer, Hamburg, Germany). Each run included a positive control (*B*. *cereus* ATCC 14579 DNA, 1 ng/μL) and negative controls (nuclease-free water and extraction blanks). Three negative controls were included per 96-well plate.

### 2.3 Conventional PCR

Conventional PCR was performed for comparison with LAMP using primers listed in **Supplementary Table 3**. Each reaction was prepared in a total volume of 25 μL containing 12.5 μL OneTaq^®^ Quick-Load^®^ 2× Master Mix (New England Biolabs, Cat# M0486), 0.2 μM each of forward and reverse primers (5 pmol each), and 2.5 μL of DNA template. The volume was adjusted to 25 μL with nuclease-free water. Thermocycling conditions were optimized for this study as follows: initial denaturation at 95°C for 15 min; 35 cycles of denaturation at 94°C for 45 s, annealing for 30 s, and extension at 72°C for 45 s; followed by a final extension at 72°C for 7 min and a hold at 4°C. Annealing temperatures were set at 63.7°C for *nheA*, 60.7°C for *cytK*, and 63°C for *entFM*. PCR products were analyzed by 1.5% agarose gel electrophoresis as described above.

### 2.4 Analytical sensitivity and specificity

*B*. *cereus* OP659027.1 was cultured overnight in LB broth at 37°C. In total, 10-fold serial dilutions (9 × 10^9^ to 9 × 10^0^ CFU/mL) were prepared in sterile PBS. Bacterial counts were confirmed by standard plate count on MYP agar (Microbiology Laboratory Guidebook [MLG], 2015). DNA was extracted from each dilution using the boiling method, and 5 μL was used for LAMP and PCR. Each dilution was tested in triplicate with three biological replicates. Pasteurized milk (confirmed *B*. *cereus*-negative by National Health and Family Planning Commission of the People’s Republic of China, 2014) was spiked with 10-fold serial dilutions of *B*. *cereus* OP659027.1 to final concentrations of 9 × 10^9^ to 9 × 10^0^ CFU/mL. DNA was extracted immediately using the boiling method and tested by LAMP and PCR as mentioned above. Purified genomic DNA from *B*. *cereus* OP659027.1 (100 ng/μL) was serially diluted 10-fold to 1 fg/μL. Each dilution (5 μL) was tested by LAMP and PCR in triplicate. Limit of detection (LOD) was defined as the lowest concentration yielding positive amplification in all replicates. Probit analysis (95% detection probability) was performed using SPSS version 25.0 (IBM, USA).

For specificity testing, all 12 *Bacillus* strains listed in **Table 1** were tested by LAMP and PCR for all three target genes. All 8 non-*Bacillus* strains were tested to assess cross-reactivity. To assess specificity in complex backgrounds, DNA from *B*. *cereus* (10³ CFU/mL) was mixed with a 10-fold excess of non-target bacteria (*E*. *coli*, *S*. *aureus*, and *Salmonella* Typhimurium) at 10^4^ CFU/mL each (*n* = 9 combinations). LAMP was performed on mixed samples to verify specific detection.

### 2.5 Field sample collection, processing, and discrepant analysis

A total of 30 field samples (5 each from layer feed, broiler feed, cattle feed, fish feed, milk, and eggs) were collected from local markets in Noakhali, Bangladesh, between January and March 2024 (**Supplementary Table 4**). Sample sizes were approximately 100 g for feeds, 50 mL for milk, and 5 whole eggs. Samples were collected aseptically in sterile containers, transported to the laboratory at 4°C, and processed within 24 h. While this sample size is adequate for preliminary validation of the LAMP assay’s performance compared to PCR, we acknowledge that larger studies will be needed for definitive diagnostic accuracy assessment.

Feed samples (1 g) were homogenized in 9 mL sterile PBS using a sterile mortar and pestle. Milk samples (1 mL) were used directly. Egg contents (1 mL) were collected after surface disinfection of shells with 70% ethanol. DNA was extracted from all samples using the boiling method (Section 2.1) without pre-culturing enrichment. All samples were coded and processed by an operator blinded to sample identity. LAMP and PCR assays were performed independently by different researchers, and results were compared only after both assays were completed. For LAMP-positive samples, viable *B*. *cereus* counts were determined by spreading 100 μL of sample homogenate on MYP agar and incubating at 37°C for 24 h. Typical pink colonies with lecithinase activity (**Supplementary Figure 1**) were counted and confirmed by Gram stain (**Supplementary Figure 2**) and biochemical tests (**Supplementary Table 5**).

For samples with discordant LAMP and PCR results, PCR inhibition testing was performed: DNA extracts from PCR-negative/LAMP-positive samples were spiked with positive control DNA (1 ng) and retested by PCR. Failure to amplify the spiked control indicated inhibition. Inhibited samples were diluted 1:5 and retested. All discrepant samples were retested after a 1:5 dilution of DNA templates to assess inhibitor effects.

### 2.6 Repeatability, reproducibility, and statistical analysis

For intra-assay repeatability, three concentrations of *B*. *cereus* DNA (100 ng, 1 ng, and 100 fg) were tested in 10 replicates within the same run. For inter-assay reproducibility, the same samples were tested on three different days by two different operators using two different heating blocks. Batch-to-batch variation was assessed by testing three different lots of WarmStart^®^ Colorimetric LAMP 2X Master Mix with standard samples.

The diagnostic performance of LAMP was evaluated using conventional PCR as the reference method. The following parameters were calculated: diagnostic sensitivity = TP/(TP+FN) × 100%; diagnostic specificity = TN/(TN+FP) × 100%; positive predictive value (PPV) = TP/(TP+FP) × 100%; negative predictive value (NPV) = TN/(TN+FN) × 100%; accuracy = (TP+TN)/(TP+TN+FP+FN) × 100%; and Cohen’s kappa coefficient (κ) with 95% confidence intervals. TP = true positive, TN = true negative, FP = false positive, and FN = false negative. Kappa values were interpreted as: <0 (no agreement), 0–0.20 (slight), 0.21–0.40 (fair), 0.41–0.60 (moderate), 0.61–0.80 (substantial), 0.81–1.00 (almost perfect). Statistical analyses were performed using SPSS version 25.0 and MedCalc version 20.0. Differences were considered significant at p < 0.05.

### 2.7 Simulated field testing

To assess field applicability, LAMP was performed using a simple water bath (accuracy ±1°C) instead of a heating block, and visual color detection without gel electrophoresis. Results were compared with laboratory-based LAMP (heating block plus gel electrophoresis). Time and cost analyses were performed comparing LAMP with conventional PCR.

## 3 Results

### 3.1 Validation of DNA extraction and primer design

The boiling method with Triton X-100 yielded DNA with a mean concentration of 38.2 ± 10.1 ng/μL and a mean A260/A280 ratio of 1.81 ± 0.09, comparable to kit-extracted DNA (45.3 ± 8.7 ng/μL and a mean ratio of 1.87 ± 0.06). No significant differences were observed in DNA yield (p = 0.12) or purity (p = 0.08). PCR inhibition (failure to amplify the spiked control) was observed in 3/30 boiling extracts (10.0%) versus 1/30 kit extracts (3.3%). All inhibited samples showed successful amplification after a 1:5 dilution. LAMP amplification success rates were 96.7% (29/30) for the boiling method versus 100% (30/30) for the kit method (p = 0.31) (**Supplementary Table 1**). These data validate the boiling method for field-compatible DNA extraction.

BLASTn analysis of all LAMP primers showed 100% identity with target sequences from 47 *B*. *cereus*, 43 *B*. *thuringiensis*, and 15 Bangladeshi *B*. *cereus* strains. No significant homology (>70% coverage, >80% identity) was observed with non-*Bacillus* foodborne pathogens. Secondary structure analysis revealed ΔG values ranging from -0.7 to -2.5 kcal/mol for hairpins and -2.8 to -5.6 kcal/mol for dimers, indicating minimal risk of non-specific structures (**Supplementary Tables 2A–D**).

### 3.2 LAMP optimization and analytical sensitivity

**Figure 1:**
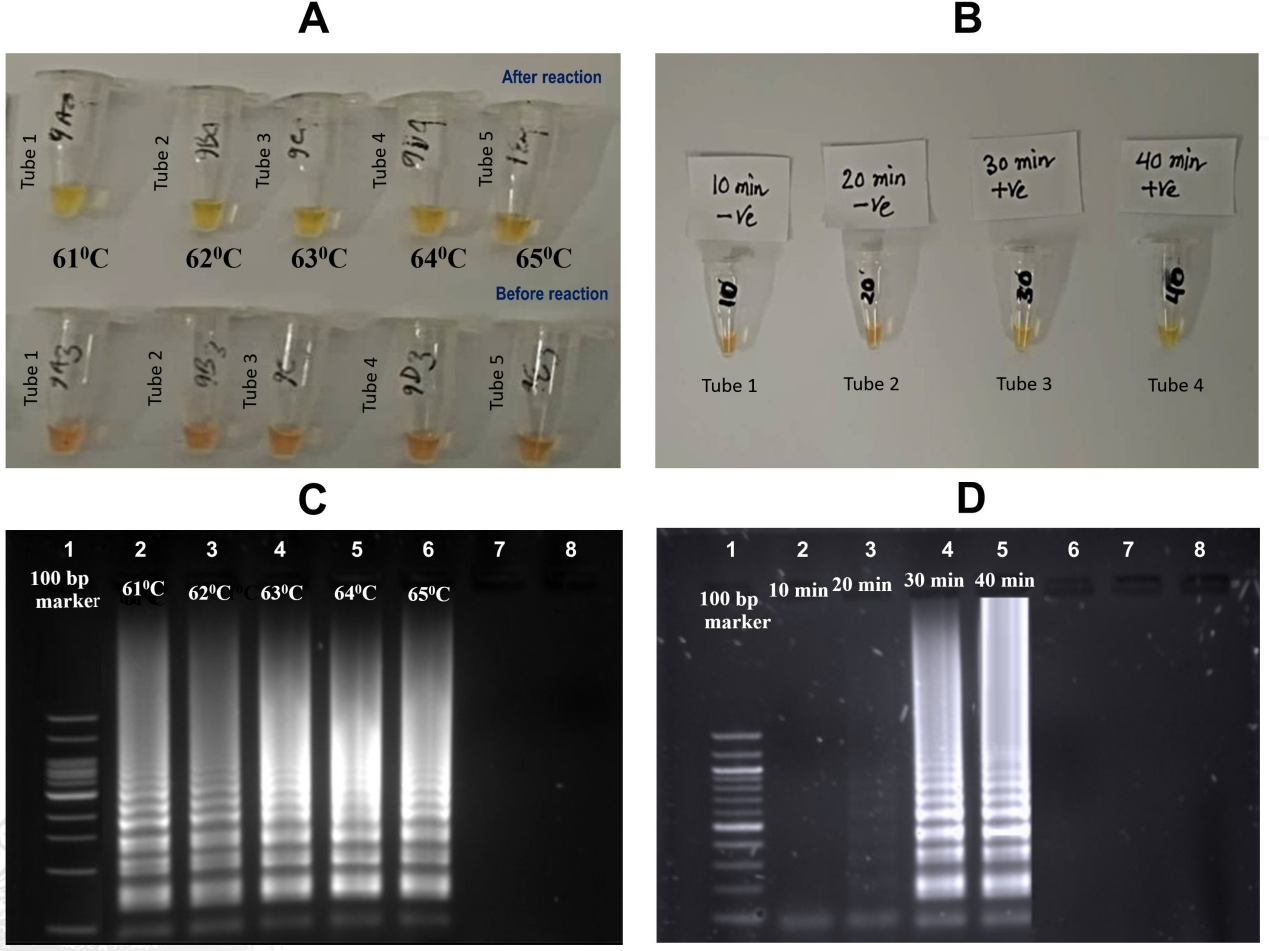
Optimization of LAMP reaction conditions. **(A)** Effect of temperature on the LAMP reaction. Tubes 1–5 show reactions before and after LAMP at 61°C, 62°C, 63°C, 64°C, and 65°C, respectively. **(B)** Effect of reaction time on the LAMP reaction. Tubes 1–4 show LAMP reactions at 10, 20, 30, and 40 min, respectively. **(C)** Gel electrophoresis of temperature optimization: Lanes 2–6 correspond to reactions at 61°C, 62°C, 63°C, 64°C, and 65°C, respectively; Lane 1: 100 bp DNA ladder. **(D)** Gel electrophoresis of time optimization: Lanes 2–5 correspond to reaction performed for 10, 20, 30, and 40 min, respectively, at 64°C; Lane 1: 100 bp DNA ladder.

LAMP reactions showed amplification at all tested temperatures (61°C–65°C), with optimal performance at 64°C. Real-time monitoring revealed the earliest threshold time at 64°C (Tt = 18.3 ± 1.2 min) compared to 63°C (Tt = 21.5 ± 1.8 min) and 65°C (Tt = 24.7 ± 2.1 min). The maximum fluorescence intensity was highest at 64°C (RFU = 12,450 ± 890) compared to the other temperatures tested (Tt range: 21.5–35.7 min; RFU range: 5,230–10,870). Gel electrophoresis showed the strongest ladder-like bands at 64°C, with a mean pixel intensity of 187.3 ± 12.4 (ImageJ) compared to 156.2 ± 15.1 at 63°C and 142.8 ± 18.3 at 65°C, confirming optimal amplification at 64°C (p < 0.05, one-way ANOVA) (**Figures 1A, C**). At 64°C, amplification was detectable at 20 min (faint bands), with optimal intensity achieved at 40 min. No significant improvement was observed beyond 40 min (**Figures 1B, D**). Positive reactions showed a distinct color change from pink/orange to yellow, visible to the naked eye. Spectrophotometric confirmation showed A420 values of 0.45–0.62 for positives versus 0.08–0.12 for negative samples.

**Figure 2:**
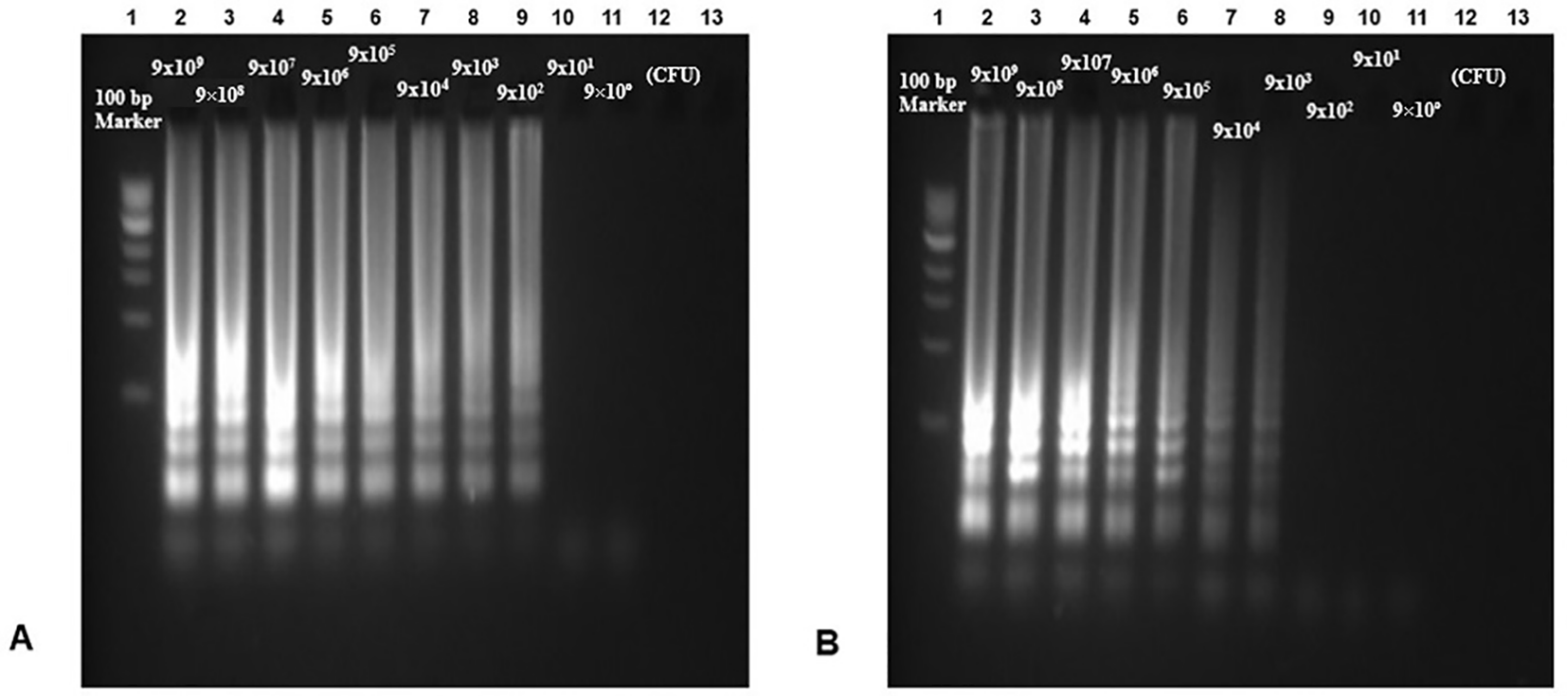
Sensitivity of the LAMP assay for culture dilutions. **(A)** Pure culture: Lanes 2–11 represent 9 × 10^9^ to 9 × 10^0^ CFU/mL (10-fold serial dilutions). Detection limit: 9 × 10² CFU/mL (Lane 9). Lower concentrations (9 × 10¹ and 9 × 10^0^ CFU/mL, Lanes 10–11) showed no amplification. Lane 1: 100 bp DNA ladder. **(B)** Spiked milk samples. Lanes 2–11 represent 9 × 10^9^ to 9 × 10^0^ CFU/mL (10-fold serial dilutions). Detection limit: 9 × 10³ CFU/mL (Lane 8). Lower concentrations (9 × 10^2^, 9 × 10^1^, and 9 × 10^0^ CFU/mL, Lanes 9–11) showed no amplification. Lane 1: 100 bp DNA ladder.

**Figure 3:**
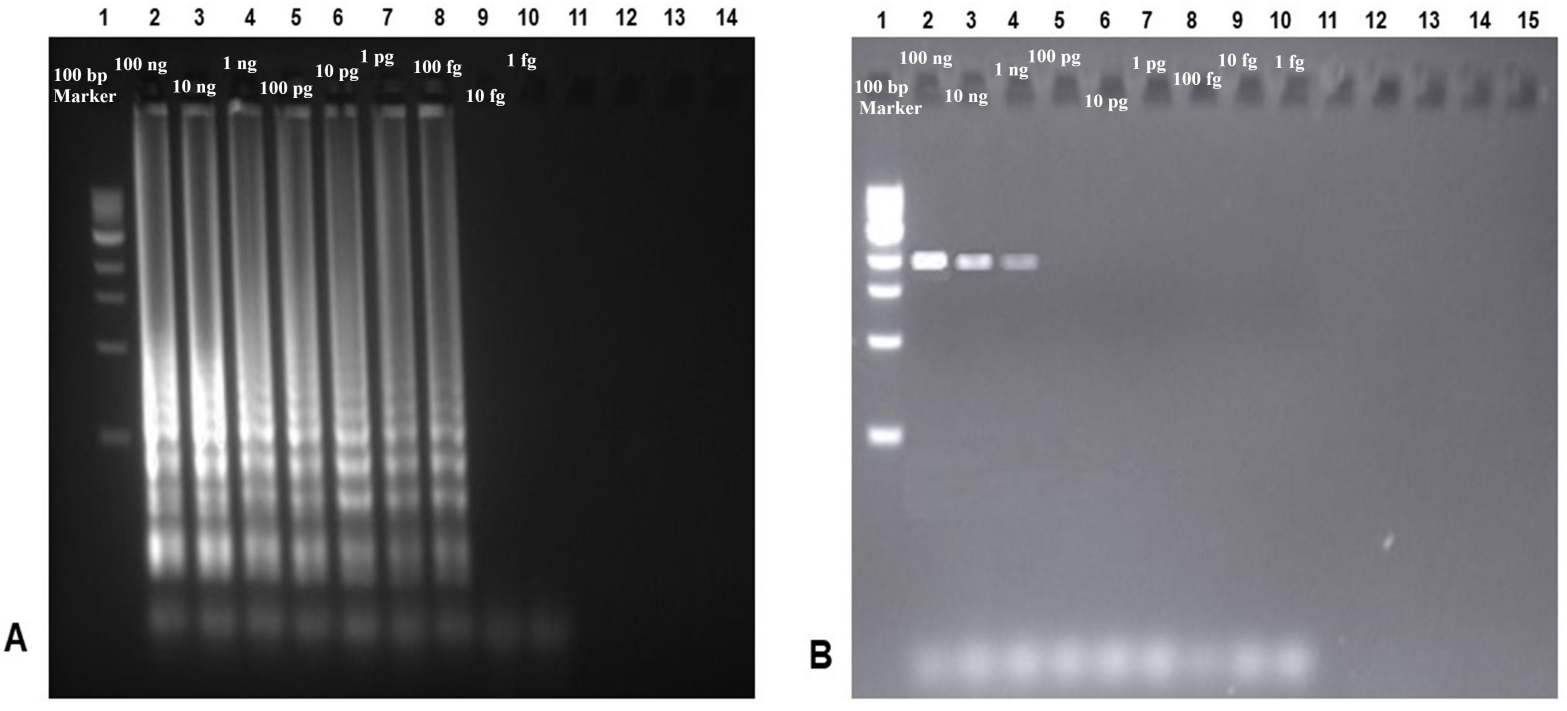
Sensitivity comparison of LAMP and PCR for pure DNA. **(A)** LAMP: Lanes 2–10 represent 100 ng to 1 fg/μL (10-fold serial dilutions). Detection limit: 100 fg/μL (Lane 8). Lane 1: 100 bp DNA Marker. **(B)** PCR (optimized conditions): Lanes 2–10 represent 100 ng to 1 fg/μL (10-fold serial dilutions). Detection limit: 1000 pg/μL (Lane 4). Lane 1: 100 bp DNA Marker.

LAMP detected *B*. *cereus* down to 9 × 10² CFU/mL in pure culture, with positive amplification in all replicates at this concentration. Lower concentrations (9 × 10¹ and 9 × 10^0^ CFU/mL) showed no amplification (**Figure 2A**). Probit analysis confirmed 95% detection probability at 8.7 × 10² CFU/mL. In the milk matrix, the LAMP detection limit was 9 × 10³ CFU/mL (95% detection probability: 8.9 × 10³ CFU/mL), representing a 10-fold reduction in sensitivity compared to pure culture due to matrix effects (**Figure 2B**). LAMP detected *B*. *cereus* DNA down to 100 fg/μL (approximately 20 genome equivalents), with 95% detection probability at 96.3 fg/μL (**Figure 3A**). Using the optimized PCR conditions (Section 2.5), the PCR detection limit was 1 ng/μL or 1000 pg/μL for pure DNA (**Figure 3B**), representing approximately 10,000-fold lower sensitivity than LAMP (100 fg/μL vs. 1000 pg/μL). For pure culture, PCR detected down to 9 × 10^4^ CFU/mL, making it 100-fold less sensitive than LAMP. LAMP LOD varied by matrix: pure culture (9 × 10² CFU/mL), milk (9 × 10³ CFU/mL), feed extract (9 × 10^4^ CFU/g), egg homogenate (9 × 10³ CFU/mL).

### 3.3 Analytical specificity

**Figure 4:**
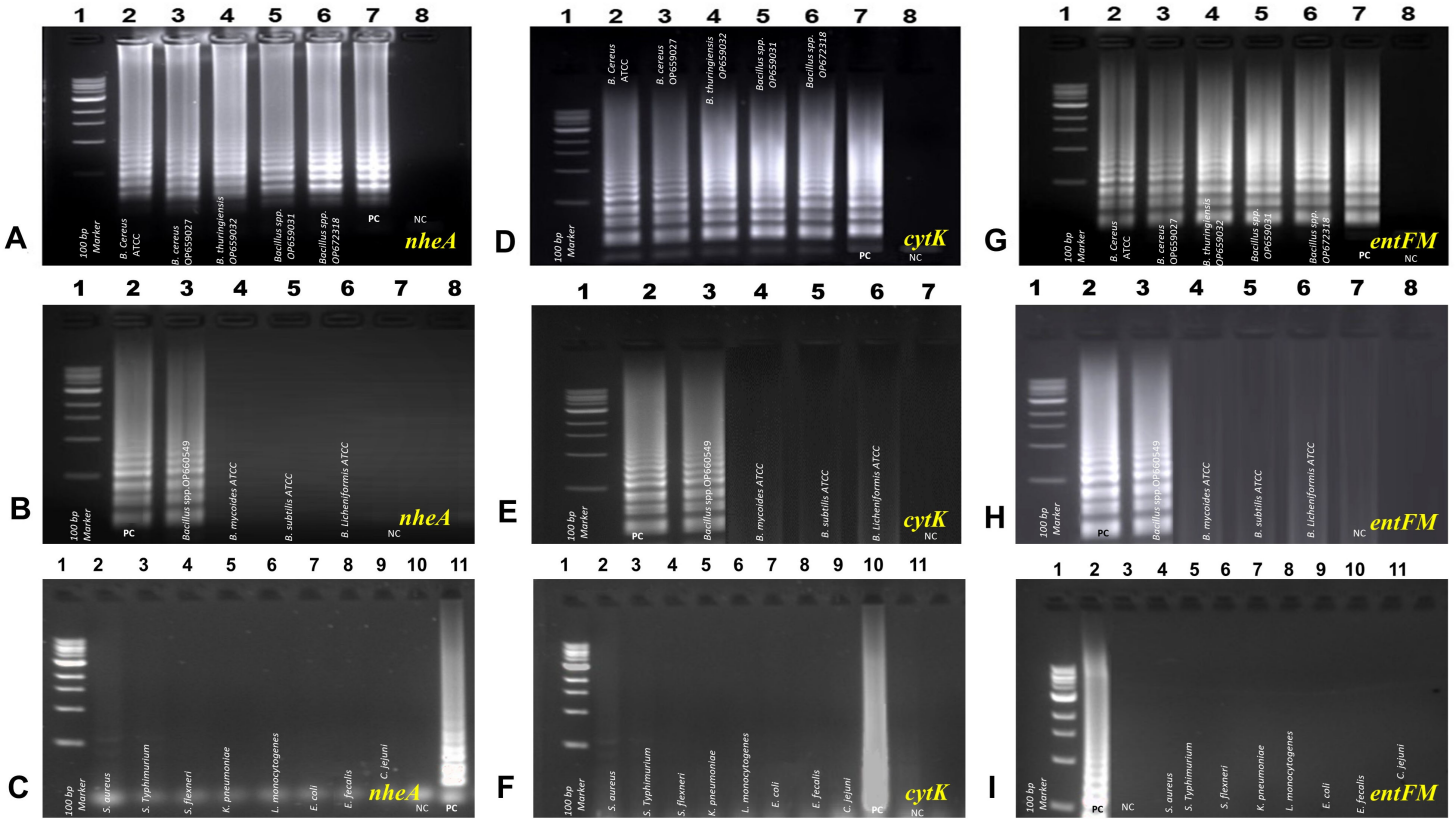
LAMP specificity results. **(A-C)** *nheA*; **(A)** *Bacillus cereus* group: Lanes 2–6 represent *B*. *cereus* ATCC 14579, *B.* cereus OP659027.1, *B*. *thuringiensis* OP659032.1, *Bacillus* spp. OP659031.1, and *Bacillus* spp. OP672318.1, respectively. Lane 7: positive control (PC). Lane 8: negative control (NC). Lane 1: 100 bp DNA ladder. **(B)** *Bacillus cereus* and other *Bacillus* groups: Lanes 3–6 represent *Bacillus* spp. OP660549.1., *B*. *mycoides* ATCC 6462, *B*. *subtilis* ATCC 6633, and *B*. *licheniformis* ATCC 14580, respectively. Lane 2: PC. Lane 7: NC. Lane 1: 100 bp DNA ladder. **(C)** Non-*Bacillus* spp. group: Lanes 2–9 represent *S*. *aureus*, *S*. Typhimurium, *S*. *flexneri*, *K*. *pneumoniae*, *L*. *monocytogenes*, *E*. *coli*, *E*. *faecalis*, and *C*. *jejuni*. Lane 10: NC. Lane 11: PC. Lane 1: 100 bp DNA ladder. **(D-F)** *cytK*; **(D)** *Bacillus cereus* group: Lanes 2–6 represent *B*. *cereus* ATCC 14579, *B*. *cereus* OP659027.1, *B*. *thuringiensis* OP659032.1, *Bacillus* spp. OP659031.1, and *Bacillus* spp. OP672318.1, respectively. Lane 7: PC. Lane 8: NC. Lane 1: 100 bp DNA ladder. **(E)** *Bacillus cereus* and other *Bacillus* groups: Lanes 3–6 represent *Bacillus* spp. OP660549.1., *B*. *mycoides* ATCC 6462, *B*. *subtilis* ATCC 6633, and *B*. *licheniformis* ATCC 14580, respectively. Lane 2: PC. Lane 7: NC. Lane 1: 100 bp DNA ladder. **(F)** Non-*Bacillus* spp. groups: Lanes 2–9 represent *S. aureus*, *S*. Typhimurium, *S*. *flexneri*, *K*. *pneumoniae*, *L*. *monocytogenes*, *E*. *coli*, *E*. *faecalis*, and *C*. *jejuni*, respectively. Lane 10: PC. Lane 11: NC. Lane 1: 100 bp DNA ladder. **(G-I)** *entFM*. **(G)** *Bacillus cereus* group: Lanes 2–6 represent *B*. *cereus* ATCC 14579, *B*. *cereus* OP659027.1, *B*. *thuringiensis* OP659032.1, *Bacillus* spp. OP659031.1, and *Bacillus* spp. OP672318.1, respectively. Lane 7: PC. Lane 8: NC. Lane 1: 100 bp DNA ladder. **(H)** *Bacillus cereus* and other *Bacillus* groups: Lanes 3–6 represent *Bacillus* spp. OP660549.1., *B*. *mycoides* ATCC 6462, *B*. *subtilis* ATCC 6633, and *B*. *licheniformis* ATCC 14580, respectively. Lane 2: PC. Lane 7: NC. Lane 1: 100 bp DNA ladder. **(I)** Non-*Bacillus* spp. group: Lanes 4–11 represent *S*. *aureus*, *S*. Typhimurium, *S*. *flexneri*, *K*. *pneumoniae*, *L*. *monocytogenes*, *E*. *coli*, *E*. *faecalis*, and *C*. *jejuni*. Lane 2: PC. Lane 3: NC. Lane 1: 100 bp DNA ladder.

**Figure 5:**
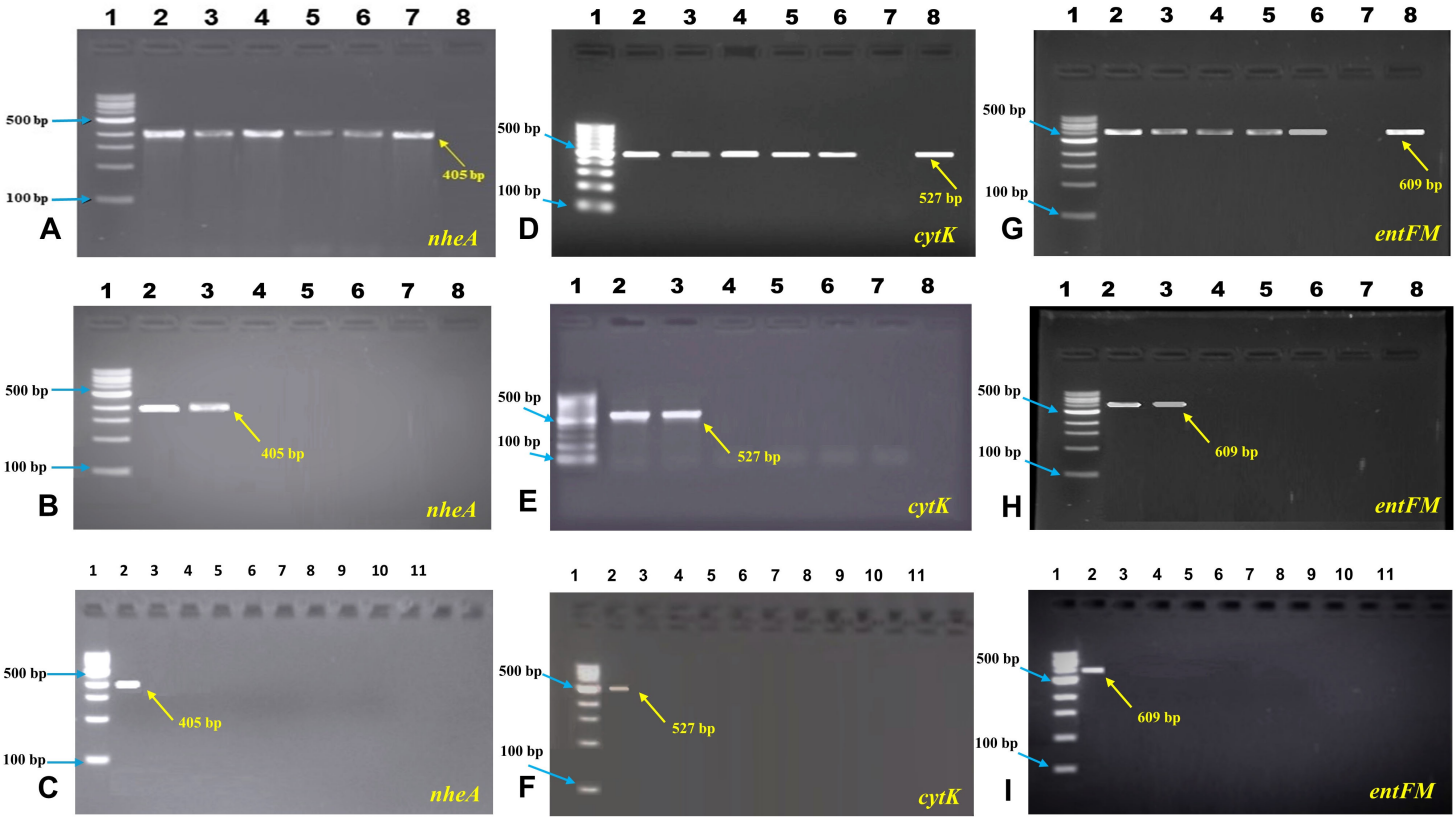
PCR specificity results. **(A–C)** *nheA*; **(A)** *Bacillus* cereus group: Lanes 2–6 represent *B. cereus* ATCC 14579, *B.* cereus OP659027.1, *B. thuringiensis* OP659032.1, *Bacillus* spp. OP659031.1, and *Bacillus* spp. OP672318.1, respectively. Lane 7: positive control (PC). Lane 8: negative control (NC). Lane 1: 100 bp DNA ladder. **(B)** *Bacillus cereus* and other Bacillus groups: Lanes 3–6 represent *Bacillus* spp. OP660549.1, *B.* mycoides ATCC 6462, *B.* subtilis ATCC 6633, and *B. licheniformis* ATCC 14580. Lane 2: PC. Lane 7: NC. Lane 1: 100 bp DNA marker. **(C)** Non-Bacillus spp. group: Lanes 4–11 represent *S. aureus*, (S) Typhimurium, *S. flexneri, K. pneumoniae, L. monocytogenes, E. coli, E. faecalis*, and *C. jejuni*, respectively. Lane 2: PC. Lane 3: NC. Lane 1: 100 bp DNA marker. **(D–F)** *cytK*; **(D)** *Bacillus cereus* group: Lanes 2–6 represent *B. cereus* ATCC 14579, *B. cereus* OP659027.1, *B. thuringiensis* OP659032.1, *Bacillus* spp. OP659031.1, and *Bacillus* spp. OP672318.1, respectively. Lane 7: NC. Lane 8: PC. Lane 1: 100 bp DNA marker. **(E)** *Bacillus cereus* and other *Bacillus* groups: Lanes 3–6 represent *Bacillus* spp. OP660549.1., *B. mycoides* ATCC 6462, *B. subtilis* ATCC 6633, and *B. licheniformis* ATCC 14580, respectively. Lane 2: PC. Lane 7: NC. Lane 1: 100 bp DNA marker. **(F)** Non-Bacillus spp. group: Lanes 3–10 represent *S. aureus*, *S.* Typhimurium, *S. flexneri, K. pneumoniae, L. monocytogenes, E. coli, E. faecalis*, and *C. jejuni.* Lane 2: PC. Lane 11: NC. Lane 1: 100 bp DNA ladder. **(G–I)** *entFM*. **(G)** *Bacillus* cereus group: Lanes 2–6 represent *B. cereus* ATCC 14579, *B. cereus* OP659027.1, *B. thuringiensis* OP659032.1, *Bacillus* spp. OP659031.1, and *Bacillus* spp. OP672318.1, respectively. Lane 7: NC. Lane 8: PC. Lane 1: 100 bp DNA ladder. **(H)** *Bacillus cereus* and other *Bacillus* groups: Lanes 3–6 represent *Bacillus* spp. OP660549.1., *B. mycoides* ATCC 6462, *B. subtilis* ATCC 6633, and *B. licheniformis* ATCC 14580, respectively. Lane 2: PC. Lane 7: NC. Lane 1: 100 bp DNA ladder. **(I)** Non-*Bacillus* spp. group: Lanes 3–10 represent *S. aureus*, S. Typhimurium, *S. flexneri, K. pneumoniae, L. monocytogenes, E. coli, E. faecalis*, and *C. jejuni*, respectively. Lane 2: PC. Lane 11: NC. Lane 1: 100 bp DNA ladder.

Among the 12 *Bacillus* strains, the *B*. *cereus* group (*B*. *cereus*, *B*. *thuringiensis*, and *B*. *cytotoxicus*) and enterotoxigenic *Bacillus* spp. tested positive for all three target genes by both LAMP and PCR. *B*. *mycoides*, *B*. *weihenstephanensis* (DSM 11821), and *B*. *anthracis* and non-cereus-group *Bacillus* species (*B*. *subtilis* and *B*. *licheniformis*) tested negative (**Table 1**; **Figures 4A, B, D, E, G, H**; **5A, B, D, E, G, H**; and **6A–F**). All eight non-Bacillus strains, including common foodborne pathogens (such as *S*. *aureus*, *E*. *coli*, *Salmonella*, and *Listeria*), tested negative for all three target genes by both methods (**Table 1**; **Figures 4C, F, I**; **5C, F, I**). LAMP successfully detected all three target genes in all mixed samples containing *B*. *cereus* (10³ CFU/mL) with a 10-fold excess of non-target bacteria (10^4^ CFU/mL each), demonstrating specific detection in complex backgrounds. No non-specific amplification was observed.

**Figure 6:**
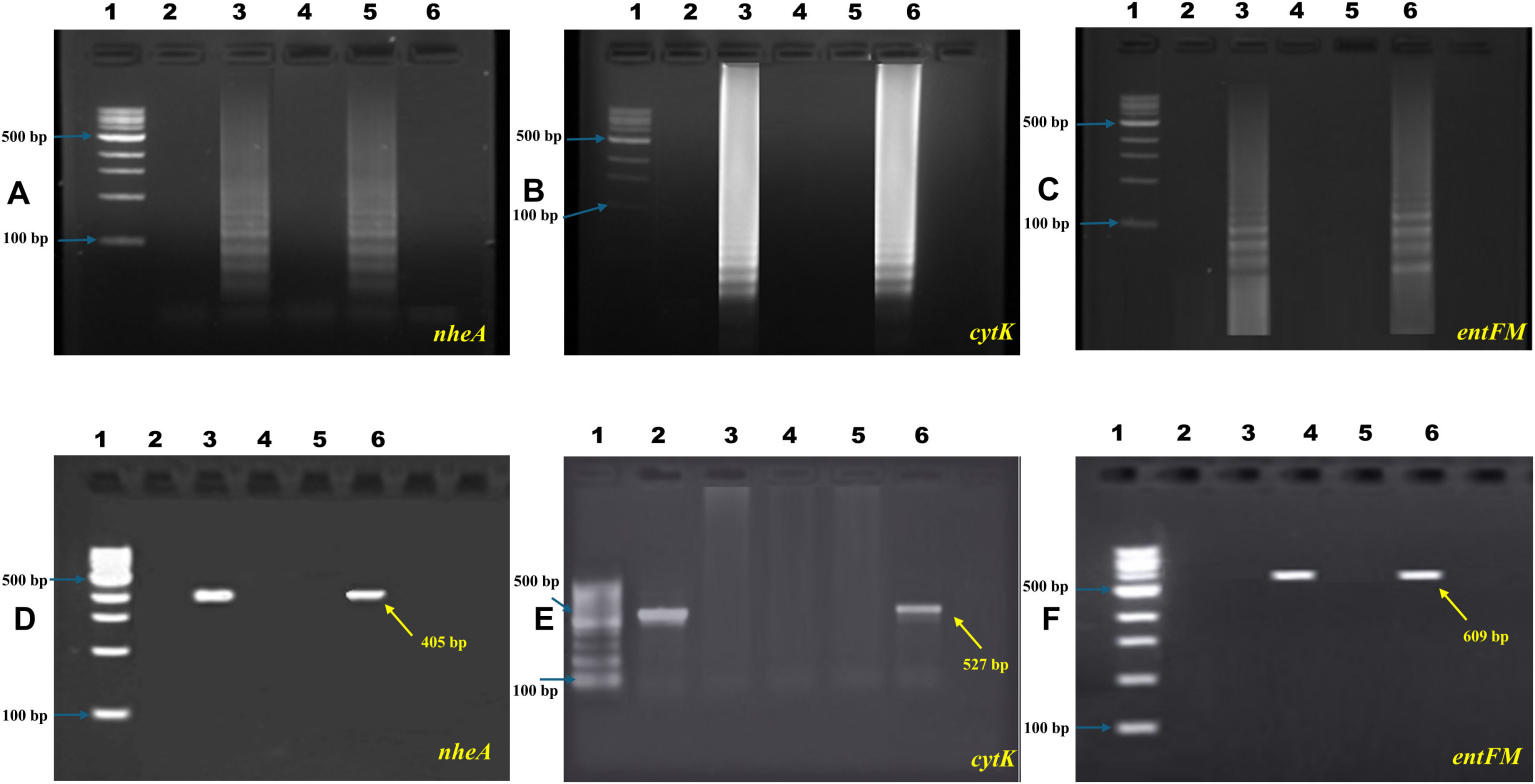
LAMP specificity results of *B*. *cereus* group members, **(A–C)**: **(A)** *nheA*; Lanes 2–4 represent *B. anthracis* ATCC 4229, *B. cytotoxicus* ATCC 10987, and *B*. *weihenstephanensis* DSM 11821, respectively. Lane 5: positive control (PC). Lane 6: negative control (NC). Lane 1: 100 bp DNA ladder. **(B)** *cytK*; Lanes 2–4 represent *B. anthracis* ATCC 4229, *B. cytotoxicus* ATCC 10987, and *B. weihenstephanensis* DSM 11821, respectively. Lane 5: NC. Lane 6: PC. Lane 1: 100 bp DNA ladder. **(C)** entFM; Lanes 2–4 represent *B. anthracis* ATCC 4229, *B*. *cytotoxicus* ATCC 10987, and *B. weihenstephanensis* DSM 11821, respectively. Lane 5: NC. Lane 6: PC. Lane 1: 100 bp DNA ladder. PCR specificity results of the *B. cereus* group member, **(D–F)**: **(D)** *nheA*; Lanes 2–4 represent *B. anthracis* ATCC 4229, *B. cytotoxicus* ATCC 10987, and *B. weihenstephanensis* DSM 11821, respectively. Lane 5: NC. Lane 6: PC. Lane 1: 100 bp DNA ladder. **(E)** *cytK*; Lanes 2–4 represent *B. cytotoxicus* ATCC 10987, *B. anthracis* ATCC 4229, and *B. weihenstephanensis* DSM 11821, respectively. Lane 5: NC. Lane 6: PC. Lane 1: 100 bp DNA ladder. **(F)** *entFM*; Lanes 2–4 represent *B. anthracis* ATCC 4229, *B. weihenstephanensis* DSM 11821, and *B. cytotoxicus* ATCC 10987, respectively. Lane 5: NC. Lane 6: PC. Lane 1: 100 bp DNA ladder.

### 3.4 Detection of enterotoxigenic *B*. *cereus* in field samples

Among the 30 field samples, LAMP detected at least one enterotoxin gene in 24 samples (80%), compared to 21 samples (70%) detected by PCR (**Table 3**). For the specific gene targets, LAMP was positive for *nheA* in 22 of 30 samples (73.3%), while PCR was positive in 19 of 30 samples (63.3%). For *cytK*, LAMP was positive in 16 of 30 samples (53.3%), compared to 12 of 30 samples (40%) by PCR. For *entFM*, LAMP detected the gene in 24 of 30 samples (80%), whereas PCR detected it in 21 of 30 samples (70%). When analyzing by sample type, among the 20 feed samples, LAMP detected *nheA* in 17 (85%), *cytK* in 12 (60%), and *entFM* in 17 (85%); PCR detected the same genes in 14 (70%), 10 (50%), and 16 (80%) samples, respectively. For the five milk samples, LAMP detected *nheA* in 2 (40%), *cytK* in 2 (40%), and *entFM* in 4 (80%); PCR detected these genes in 2 (40%), 1 (20%), and 2 (40%) samples, respectively. Regarding the five egg samples, LAMP detected *nheA* in 3 (60%), *cytK* in 2 (40%), and *entFM* in 3 (60%); PCR detected these genes in 3 (60%), 1 (20%), and 3 (60%) samples, respectively. For LAMP-positive samples, viable *B*. *cereus* counts ranged from 2.3 × 10² to 4.7 × 10^5^ CFU/g (feed samples) and from 1.8 × 10¹ to 6.2 × 10³ CFU/mL (milk and egg samples). All samples with counts >10³ CFU/g or CFU/mL were LAMP-positive, while samples with counts <10² CFU/g or CFU/mL showed variable LAMP results, consistent with the assay’s LOD.

**Table 3:** Analysis of field samples for enterotoxigenic *Bacillus* spp. by LAMP and conventional PCR.

Sample		LAMP			PCR		
S/N	Type	*nheA*	*cytK*	*entFM*	*nheA*	*cytK*	*entFM*
1	LF	+	+	+	+	+	+
2		+	–	+	+	–	+
3		+	+	+	+	+	+
4		+	+	+	+	+	+
5		+	–	+	+	–	+
Total LF (*n* = 5)	Positive (%)	5 (100)	3 (60)	5 (100)	5 (100)	3 (60)	5 (100)
6	BF	+	+	+	+	+	+
7		+	+	+	+	+	+
8		+	+	+	–	+	+
9		+	+	+	+	–	+
10		+	–	+	–	–	+
Total BF (*n* = 5)	Positive (%)	5 (100)	4 (80)	5 (100)	3 (60)	3 (60)	5 (100)
11	CF	+	+	+	+	+	+
12		+	+	+	–	–	+
13		+	–	+	+	–	–
14		–	–	–	–	–	–
15		–	–	–	–	–	–
Total CF (*n* = 5)	Positive (%)	3 (60)	2 (40)	3 (60)	2 (40)	1 (20)	2 (40)
16	FF	+	+	+	+	+	+
17		+	+	+	+	+	+
18		+	+	+	+	+	+
19		–	–	–	–	–	–
20		+	–	+	+	–	+
Total FF (*n* = 5)	Positive (%)	4 (80)	3 (60)	4 (80)	4 (80)	3 (60)	4 (80)
Total feed (*n* = 20)		17 (85)	12 (60)	17 (85)	14 (70)	10 (50)	16 (80)
21	M	–	–	+	+	–	+
22		+	+	+	+	+	+
23		–	–	–	–	–	–
24		+	–	+	–	–	–
25		–	+	+	–	–	–
Total M (*n* = 5)	Positive (%)	2 (40)	2 (40)	4 (80)	2 (40)	1 (20)	2 (40)
26	E	+	+	+	+	–	+
27		+	+	–	+	+	+
28		–	–	+	–	–	+
29		+	–	+	+	–	–
30		–	–	–	–	–	–
Total E (*n* = 5)	Positive (%)	3 (60)	2 (40)	3 (60)	3 (60)	1 (20)	3 (60)
Total Sample (*n* = 30)		22 (73.3)	16 (53.3)	24 (80)	19 (63.3)	12 (40)	21 (70)

LL, Layer feed; BF, Broiler feed; CF, Cattle feed; FF, Fish feed; M, Milk; E, Egg.

### 3.5 Diagnostic performance and discrepancy analysis

Among a total of 90 tests (30 samples × 3 genes), LAMP detected 62 positive results (*nheA* = 22, *cytK* = 16, and *entFM* = 24), whereas PCR detected 52 positive results (*nheA* = 19, *cytK* = 12, and *entFM* = 21). The confusion matrix summarized 49 true positives, 26 true negatives, 13 false positives, and 2 false negatives. LAMP showed high sensitivity for *nheA* (94.7%, 95% CI: 74.0%–99.9%), *cytK* (100%, 95% CI: 71.5%–100%), and *entFM* (95.2%, 95% CI: 76.2%–99.9%), with an overall sensitivity of 96.1% (95% CI: 86.5%–99.5%). Specificity was 63.6% for *nheA*, 73.7% for *cytK*, and 55.6% for *entFM*, with an overall specificity of 66.7% (95% CI: 49.8%–80.9%). Overall accuracy was 83.3%, and Cohen’s kappa was 0.62 (95% CI: 0.48–0.76), indicating substantial agreement between LAMP and PCR (**Table 4**; **Supplementary Table 6**). A total of 15 discrepancies (16.7%) were observed, predominantly LAMP-positive/PCR-negative (13/15, 86.7%), occurring most frequently in milk (5 discrepancies) and broiler feed (4 discrepancies) samples. The two false negatives (LAMP-negative/PCR-positive) occurred in one milk sample for *nheA* (sample 21) and one egg sample for *entFM* (sample 27), both with low bacterial loads (<10² CFU/mL). Upon retesting with concentrated DNA (10 μL instead of 5 μL), both became LAMP-positive, indicating that these samples were near the limit of detection, where stochastic effects may occur. Investigation of the LAMP-positive/PCR-negative discrepancies revealed evidence of PCR inhibition. After 1:5 dilution, 10 of the 13 inhibited samples became PCR-positive, confirming the presence of inhibitory substances in these matrices.

**Table 4:** Diagnostic performance of LAMP compared with conventional PCR (per-gene analysis based on 90 total tests).

Parameter	*nheA*	*cytK*	*entFM*	Overall (95% CI)
TP (LAMP+/PCR+)	18	11	20	49
FP (LAMP+/PCR–)	4	5	4	13
FN (LAMP–/PCR+)	1	0	1	2
TN (LAMP–/PCR–)	7	14	5	26
Total tests	30	30	30	90
Sensitivity (%)	94.7 (74.0-99.9)	100 (71.5-100)	95.2 (76.2-99.9)	96.1 (86.5-99.5)
Specificity (%)	63.6 (30.8-89.1)	73.7 (48.8-90.9)	55.6 (21.2-86.3)	66.7 (49.8-80.9)
PPV (%)	81.8	68.8	83.3	79.0
NPV (%)	87.5	100	83.3	92.9
Accuracy (%)	83.3	83.3	83.3	83.3
Kappa (κ)	0.61 (0.36-0.86)	0.70 (0.47-0.93)	0.54 (0.28-0.80)	0.62 (0.48-0.76)

TP, true positive; FP, false positive; FN, false negative; TN, true negative; PPV, positive predictive value; NPV, negative predictive value.

### 3.6 Repeatability, reproducibility, and field applicability

Intra-assay repeatability showed that the coefficient of variation (CV) for Tt values ranged from 2.3% to 4.7% across the three concentrations (100 ng: 2.3%; 1 ng: 3.1%; and 100 fg: 4.7%), indicating excellent repeatability. Inter-assay reproducibility showed that CVs across three days, two operators, and two heating blocks ranged from 3.8% to 6.2% (day-to-day: 4.2%; operator-to-operator: 5.1%; and instrument-to-instrument: 4.8%), demonstrating good inter-assay reproducibility. No significant differences in Tt values (p > 0.05, ANOVA) or detection limits were observed across the three master-mix lots.

For simulated field testing, LAMP performed using a simple water bath showed 93.3% agreement (84/90 tests) with laboratory-based LAMP (heating block plus gel electrophoresis). All discrepancies occurred at concentrations near the LOD. Colorimetric results interpreted by the naked eye showed 94.4% agreement with gel electrophoresis (κ = 0.88). Discrepancies were primarily weak positives (score +) misclassified as negative. The time- and cost-benefit analysis is presented in **Supplementary Table 7**.

## 4 Discussion

Food- and feed-borne *B*. *cereus* is a causative agent of a variety of foodborne illnesses causing diarrhea in both humans and animals (Sultana et al., 2017; Habib et al., 2021; Nupur et al., 2023). This study reports the first validated LAMP assay for the detection of enterotoxigenic *B*. *cereus* in food and feed samples from Bangladesh, targeting three major enterotoxin genes (*nheA*, *cytK*, and *entFM*). The method demonstrated high sensitivity (LOD: 100 fg DNA, 9 × 10² CFU/mL in pure culture), excellent specificity for *B*. *cereus* group members, and good diagnostic performance against conventional PCR (sensitivity 96.1%, specificity 66.7%, κ = 0.62). The assay’s simplicity, speed (90 min), and minimal equipment requirements make it particularly suitable for resource-limited settings where food safety surveillance infrastructure is limited.

Previous LAMP assays for *B*. *cereus* have targeted individual genes, including *16S rDNA* (Liu et al., 2011), *nheB* (Busch et al., 2022), *cytK*, *entFM* (Mandappa et al., 2016), and *ces* (emetic toxin) (Wang et al., 2022). Our study extends this work by simultaneously validating three enterotoxin genes (*nheA*, *cytK*, and *entFM*) targeting major diarrheal virulence determinants. The newly designed *nheA* primers showed robust performance, with in silico specificity for *B*. *cereus* group members and experimental validation against 12 *Bacillus* strains.

The sensitivity achieved (100 fg DNA) compares favorably with previously reported LAMP assays for *B*. *cereus* (**Table 5**). The 10-fold reduction in sensitivity observed in the milk matrix (9 × 10³ CFU/mL) compared with pure culture (9 × 10² CFU/mL) is consistent with matrix effects reported by others (Mandappa et al., 2016; Wang et al., 2022) and likely reflects interference from milk proteins and fats during DNA extraction and amplification. The infectious dose for *B*. *cereus* diarrheal syndrome is typically >10^5^–10^7^ CFU/g of food (EFSA, 2016). Our LOD of 9 × 10² CFU/mL in pure culture and 9 × 10³ CFU/mL in milk is therefore well below the clinically relevant threshold, providing a safety margin for early detection before toxin production reaches hazardous levels.

**Table 5:** Comparison of LAMP sensitivity with previous studies.

Study	Target gene(s)	LOD (pure DNA)	LOD (pure culture)	LOD (food matrix)
This study	*nheA*, *cytK*, *entFM*	100 fg/μL	9×10² CFU/mL	9×10³ CFU/mL (milk)
Liu et al., 2011	*cesA*	500 fg/μL	10³ CFU/mL	Not tested
Mandappa et al., 2016	*cytK*, *entFM*	200 fg/μL	5×10² CFU/mL	10^4^ CFU/mL (milk)
Busch et al., 2022	*nheB*	50 fg/μL	10² CFU/mL	10³ CFU/mL (cheese)
Wang et al., 2022	*cesA*, 16S rDNA	100 fg/μL	5×10² CFU/mL	10³ CFU/mL (dairy)

The LAMP assay showed high overall sensitivity of 96.1% (95% CI: 86.5%–99.5%) compared with PCR, with gene-specific sensitivities of 94.7% for *nheA*, 100% for *cytK*, and 95.2% for *entFM*. Two false negatives (one each for *nheA* and *entFM*) were observed in samples with very low target DNA concentration (<10¹ CFU/mL equivalent) near the analytical limit of LAMP. Specificity was 66.7% overall (95% CI: 49.8%–80.9%), reflecting 13 LAMP-positive/PCR-negative cases. This apparent “false positivity” was largely attributable to PCR inhibition in complex matrices (milk and feed), as confirmed by spiked control experiments. After dilution of inhibited samples, 10 of 13 became PCR-positive, indicating that LAMP’s greater tolerance to inhibitors (due to Bst polymerase robustness) allowed detection where PCR failed. The remaining three discrepant samples had bacterial loads below 10² CFU/mL, near the PCR detection limit. These findings align with previous reports that LAMP can outperform PCR in inhibitor-rich food matrices, although occasional false negatives may occur near the detection threshold (Notomi et al., 2000; Wong et al., 2018). The substantial agreement (κ = 0.62, 95% CI: 0.48–0.76) supports LAMP as a reliable alternative, particularly for screening purposes where high sensitivity is prioritized.

The assay detected all tested *B*. *cereus* group members (*B*. *cereus*, *B*. *thuringiensis*, and *B. cytotoxicus*) but not non-cereus group Bacillus or non-Bacillus pathogens. Importantly, *B*. *weihenstephanensis* and *B*. *anthracis* tested negative for all three targets, as these species lack the specific enterotoxin genes. This broad detection within the *B*. *cereus* group is expected due to genetic relatedness and the presence of enterotoxin genes across the group (Ngamwongsatit et al., 2008; Cardazzo et al., 2008).

While *B*. *thuringiensis* is generally considered less pathogenic than *B*. *cereus*, strains harboring enterotoxin genes have been associated with foodborne outbreaks (McIntyre et al., 2008). Detection of these strains by our assay is therefore relevant for food safety, as they may pose similar risks. The broad detection within enterotoxigenic *B*. *cereus* group members is appropriate for a food safety screening assay, although species-level differentiation would require additional testing (e.g., microscopy for parasporal crystals or species-specific PCR) (Ogawa et al., 2015; Busch et al., 2022).

The LAMP assay developed here addresses critical needs for food safety monitoring in Bangladesh and similar resource-limited settings:

• Rapid results: 90 min from sample to answer vs. 3–5 days for culture-based methods.
• Simple equipment: Only a water bath or heating block is required; no thermal cycler is needed.
• Visual detection: No specialized training is required for result interpretation.
• Low cost: USD 2.50 per sample vs. USD 5.80 per sample for PCR.
• No culture pre-enrichment: Direct testing from sample lysates reduces time and complexity.

These characteristics make the assay suitable for deployment at various levels:

• Central laboratories: For confirmatory testing and outbreak investigations.
• Regional food testing centers: For routine surveillance of high-risk foods.
• Mobile testing units: For field investigations during suspected outbreaks.
• Food processing facilities: For on-site quality control.

The high prevalence of enterotoxin genes detected in this study (80% of samples positive by LAMP) underscores the importance of routine surveillance. Feed contamination (100% positive) is particularly concerning, as contaminated feed can introduce *B*. *cereus* into the animal food chain, ultimately affecting human consumers through meat, milk, and eggs (Haque et al., 2022). Milk and egg contamination (80% positive) represent direct consumer exposure pathways requiring urgent attention.

## 5 Limitations and future directions

This study has several limitations that should be addressed in future work.

First, the assay cannot distinguish *B*. *cereus* from closely related *B*. *thuringiensis* or other *B*. *cereus* group members. Future development of a two-step strategy (group-specific detection followed by species-specific confirmation) or the inclusion of species-specific targets could address this limitation. Second, the current endpoint LAMP format provides only qualitative results. The implementation of real-time LAMP using portable fluorometers would enable quantification, which is valuable for risk assessment and determining whether contamination levels exceed regulatory thresholds (typically >10^5^ CFU/g for *B*. *cereus* in food). Third, our method detects DNA from both vegetative cells and spores, but the extraction efficiency from spores may be lower due to their resistant structure. Incorporation of spore lysis steps (e.g., enzymatic pretreatment with lysozyme and mechanical disruption with bead-beating) could improve detection of spore-contaminated samples, which is critical because spores are the primary form of *B*. cereus contamination in many foods. Fourth, although three genes were targeted individually, a multiplex LAMP would be more efficient for routine screening. Development of a multiplex LAMP with gene-specific probes or melt-curve analysis should therefore be a priority for future work. Fifth, the relatively small sample size (*n* = 30) limits generalizability of our findings. Larger studies involving geographically diverse samples from different regions of Bangladesh and other countries are needed to confirm assay performance. Additionally, *Bacillus pseudomycoides*—a closely related member of the *B. cereus* group that may harbor enterotoxin genes—was not available for testing in this study. Future work should include this species to fully characterize the inclusivity of the LAMP assay across the entire *B. cereus* group. External validation in independent laboratories would also strengthen confidence in the method. Finally, the absence of comparison with quantitative real-time PCR (qPCR) limits benchmarking against gold-standard molecular methods. Future studies should include qPCR comparisons to fully characterize LAMP performance. Although we validated the assay using milk, feed, and eggs, its performance in other matrices, such as meat, fish, and ready-to-eat foods, should be evaluated.

## 6 Conclusion

This study establishes the first validated LAMP assay for the detection of enterotoxigenic *B*. *cereus* in food and feed samples from Bangladesh, targeting three major enterotoxin genes (*nheA*, *cytK*, and *entFM*). This assay was conducted under optimized reaction conditions at 64°C for 40 min, enabling rapid detection within 90 min from sample to result. Analytical sensitivity assessment revealed a LOD of 100 fg of genomic DNA, 9 × 10² CFU/mL in pure culture, and 9 × 10³ CFU/mL in spiked milk samples, representing approximately 10,000-fold greater sensitivity than conventional PCR. Specificity testing demonstrated 100% detection of all tested *B*. *cereus* group members, with no cross-reactivity with 13 non-target foodborne pathogens. Diagnostic performance evaluation using field samples yielded a sensitivity of 96.1% and a specificity of 66.7%, with substantial agreement with PCR (κ = 0.62). The assay proved suitable for field sample applications due to its simple equipment requirements, visual detection capability, and low cost (US$2.50 per sample), in addition to its ability to test directly from sample lysates without the need for culture enrichment. Practical utility was confirmed by successful detection of the target genes in 73.3%-80% of field samples (depending on the gene), highlighting the assay’s utility for routine surveillance of enterotoxigenic *B*. *cereus*.

## Data Availability

The datasets presented in this study can be found in online repositories. The names of the repository/repositories and accession number(s) can be found in the article/**Supplementary Material**.
