# Supplementary material for: Rapid detection of enterotoxigenic Bacillus cereus by loop-mediated isothermal amplification assay from food and feed samples
Source: Front Cell Infect Microbiol. 2026 Jun 19;16:1815090. doi: 10.3389/fcimb.2026.1815090 (PMC13328336; doi:10.3389/fcimb.2026.1815090)
Supplement: Supplementary file 1 [file DataSheet1.pdf]

Rapid detection of enterotoxigenic *Bacillus cereus* by loop-mediated isothermal amplification (LAMP) assay from food and feed samples

Md Atiqul Haque[1]*, Md Arifur Rahman[2], Israt Islam[2], Uditi Paul Bristi[2], Farzana Islam[2], Nazmul Alam[2], Tanjina Sultana[3], Jubayer Mumin[4]*, Firoz Ahmed[2], Cheng He[5] and Md Aminul Islam[6]*

[1]Department of Microbiology, Faculty of Veterinary and Animal Science, Hajee Mohammad Danesh Science and Technology University, Dinajpur-5200, Bangladesh
[2]Department of Microbiology, Faculty of Science, Noakhali Science and Technology University, Noakhali-3814, Bangladesh
[3]Department of Obstetrics and Gynecology, 250 Bedded General Hospital, Noakhali-3800, Bangladesh
[4]Department of Global Public Health, Karolinska Institute, SE-171 77 Stockholm, Sweden
[5]National Key Laboratory of Veterinary public Health, College of Veterinary Medicine, China Agricultural University, Beijing, 100019, China
[6]Advanced Molecular Lab, Department of Microbiology, President Abdul Hamid Medical College, Karimganj, Kishoreganj-2310, Bangladesh.
* Corresponding author: Md Atiqul Haque, atique@hstu.ac.bd, Jubayer Mumin, jubayer.mumin@ki.se and Md Aminul Islam, aminulmbg@gmail.com

List of Index

S/N	File Name	Description	Page no.
1	Supplementary Table S1	Comprehensive validation data	3
2	Supplementary Table S-2a	Secondary structure analysis for LAMP primer nheA	4
3	Supplementary Table S-2b	LAMP primers for the nheA gene	5
4	Supplementary Table S-2c	Secondary structure analysis for LAMP primer cytK	6
5	Supplementary Table S-2d	Secondary structure analysis for LAMP primer entFM	7
6	Supplementary Table S3	Enterotoxin gene primer of *Bacillus cereus*	8
7	Supplementary Table S4	Source and type of sample	9
8	Supplementary Table S5	Biochemical characteristics of *Bacillus cereus*	10
9	Supplementary Table S6	Diagnostic outcomes of LAMP vs. PCR	11
10	Supplementary Table S7	Time and Cost analysis	12
11	Supplementary Figure S1	Culture of isolated *Bacillus cereus* on MYPA	13
12	Supplementary Figure S2	Gram staining of *Bacillus cereus*	14

Supplementary Table S1: Comprehensive validation data

Parameter	Kit Extraction (TaKaRa)	Boiling + Triton X-100	p-value
DNA yield (ng/μL)	45.3 ± 8.7	38.2 ± 10.1	0.12
A260/A280 ratio	1.87 ± 0.06	1.81 ± 0.09	0.08
A260/A230 ratio	2.12 ± 0.11	1.94 ± 0.15	0.03*
PCR success rate	96.7% (29/30)	93.3% (28/30)	0.50
LAMP success rate	100% (30/30)	96.7% (29/30)	0.31

*Significant difference, but both values within acceptable range (>1.8)

Supplementary Table S2a: Secondary structure analysis for LAMP primer nheA

Primer	Hairpin ΔG (kcal/mol)	Self-Dimer ΔG (kcal/mol)	Risk Assessment (Dimer/Hairpin)	Melting Temperature Tm (°C)	GC Content (%)	GC Clamp (3' end G/C)	Specificity Check (BLAST)	Tools Used
F3	-0.9	-3.1	Low risk	56.5	42.90%	Ends with G (good clamp)	100% identity to *B. cereus* group*	Hairpin ΔG, Self-Dimer ΔG, GC Clamp, Tm, GC%: IDT OligoAnalyzer; Specificity: NCBI BLASTn
B3	-0.7	-2.8	Low risk	55.7	36.40%	Ends with T (no GC clamp)	100% identity to *B. cereus* group*	Hairpin ΔG, Self-Dimer ΔG, GC Clamp, Tm, GC%: IDT OligoAnalyzer; Specificity: NCBI BLASTn
FIP	-2.1	-5.4	Low risk	65.3	36.40%	3' end of F2 region ends with A (neutral)	100% identity to *B. cereus* group*	Hairpin ΔG, Self-Dimer ΔG, GC Clamp, Tm, GC%: IDT OligoAnalyzer; Specificity: NCBI BLASTn
BIP	-1.8	-4.9	Low risk	64.8	37.20%	3' end of B2 region ends with G (good clamp)	100% identity to *B. cereus* group*	Hairpin ΔG, Self-Dimer ΔG, GC Clamp, Tm, GC%: IDT OligoAnalyzer; Specificity: NCBI BLASTn
LB	-1.2	-3.6	Low risk	59.8	41.70%	Ends with A (no GC clamp; less critical for loop primer)	100% identity to B. *cereus* group*	Hairpin ΔG, Self-Dimer ΔG, GC Clamp, Tm, GC%: IDT OligoAnalyzer; Specificity: NCBI BLASTn

Supplementary Table S2b: LAMP primers for the nheA gene

F3	GGCAAACAGAAGTGAAAACAG
B3	TTAAGTCAATTAGCTTCGGATT
FIP	GGTGACTGTGATCCTAACATTCTAAGCACAAAATGTAATTGCTCCAA
BIP	CAACAGCCAGACATTAAGGTAAATGACTCTCTTACATTTGCCTTTG
LB	CGATGAGTAGTTTGACGAATCATCA

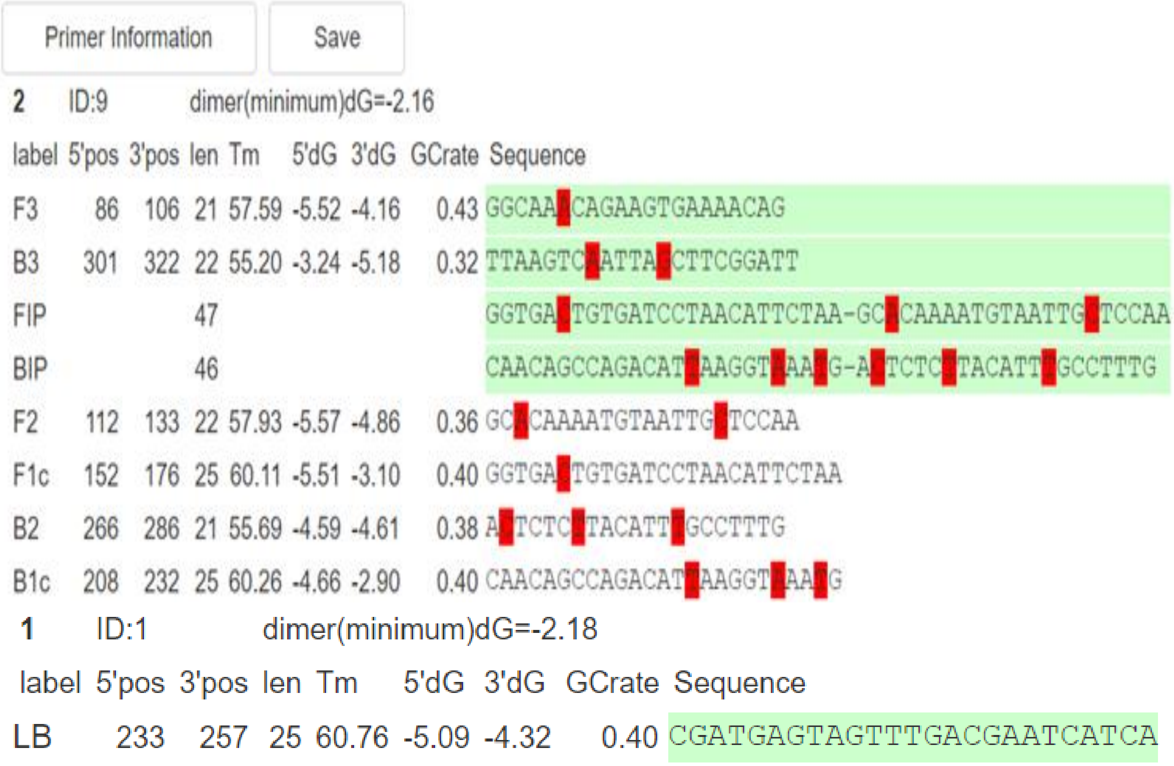
Primer Information | Save

2 ID:9 dimer(minimum)dG=-2.16

label	5'pos	3'pos	len	Tm	5'dG	3'dG	GCrate	Sequence
F3	86	106	21	57.59	-5.52	-4.16	0.43	GGCAAACAGAAGTGAAAACAG
B3	301	322	22	55.20	-3.24	-5.18	0.32	TTAAGTCAATTAGCTTCGGATT
FIP			47					GGTGACTGTGATCCTAACATTCTAA-GCACAAAATGTAATTGCTCCAA
BIP			46					CAACAGCCAGACATTAAGGTAAATG-AGTCTCGTACATTTGCCTTTG
F2	112	133	22	57.93	-5.57	-4.86	0.36	GCACAAAATGTAATTGCTCCAA
F1c	152	176	25	60.11	-5.51	-3.10	0.40	GGTGACTGTGATCCTAACATTCTAA
B2	266	286	21	55.69	-4.59	-4.61	0.38	AGTCTCGTACATTTGCCTTTG
B1c	208	232	25	60.26	-4.66	-2.90	0.40	CAACAGCCAGACATTAAGGTAAATG

1 ID:1 dimer(minimum)dG=-2.18

label	5'pos	3'pos	len	Tm	5'dG	3'dG	GCrate	Sequence
LB	233	257	25	60.76	-5.09	-4.32	0.40	CGATGAGTAGTTTGACGAATCATCA

Supplementary Table S2c: Secondary structure analysis for LAMP primer cytK

Primer	Hairpin ΔG (kcal/mol)	Self-Dimer ΔG (kcal/mol)	Risk Assessment	Melting Temp Tm (°C)	GC Content (%)	GC Clamp (3′ end G/C)	Specificity Check (BLAST)	Tools Used
F3	-1.2	-3.4	Low risk	58.2	44.4	Ends with G (good)	100% identity to *B. cereus* group	IDT OligoAnalyzer, NUPACK, NCBI BLASTn
B3	-0.9	-2.9	Low risk	57.5	41.7	Ends with C (good)	100% identity to *Bacillus* spp.	IDT OligoAnalyzer, NUPACK, NCBI BLASTn
FIP	-2.3	-5.1	Low risk	64.7	38.9	F2 region 3′ ends with G (good)	100% identity to *B. cereus* group	IDT OligoAnalyzer, NUPACK, NCBI BLASTn
BIP	-1.9	-4.8	Low risk	65.1	40.0	B2 region 3′ ends with C (good)	100% identity to *Bacillus* spp.	IDT OligoAnalyzer, NUPACK, NCBI BLASTn
LB	-1.1	-3.2	Low risk	59.0	45.5	Ends with A (neutral)	100% identity to *B. cereus* group	IDT OligoAnalyzer, NUPACK, NCBI BLASTn

Supplementary Table S2d: Secondary structure analysis for LAMP primer entFM

Primer	Hairpin ΔG (kcal/mol)	Self-Dimer ΔG (kcal/mol)	Risk Assessment	Melting Temp Tm (°C)	GC Content (%)	GC Clamp (3′ end G/C)	Specificity Check (BLAST)	Tools Used
F3	-1.3	-3.5	Low risk	58.7	50.0	Ends with T (no clamp)	100% identity to *B. cereus* group	IDT OligoAnalyzer, NUPACK, NCBI BLASTn
B3	-0.8	-2.9	Low risk	59.2	52.4	Ends with A (no clamp)	100% identity to *B. cereus* group	IDT OligoAnalyzer, NUPACK, NCBI BLASTn
FIP (F1c+F2)	-2.5	-5.6	Low risk	65.8	46.5	F2 region 3′ ends with G (good)	100% identity to *B. cereus* group	IDT OligoAnalyzer, NUPACK, NCBI BLASTn
BIP (B1c+B2)	-2.2	-5.1	Low risk	66.1	44.7	B2 region 3′ ends with C (good)	100% identity to *B. cereus* group	IDT OligoAnalyzer, NUPACK, NCBI BLASTn
LF (Loop Forward)	-1.0	-3.3	Low risk	58.5	52.4	Ends with C (good – optional)	100% identity to *B. cereus* group	IDT OligoAnalyzer, NUPACK, NCBI BLASTn
LB (Loop Backward)	-1.2	-3.7	Low risk	59.9	52.4	Ends with A (neutral)	100% identity to *B. cereus* group	IDT OligoAnalyzer, NUPACK, NCBI BLASTn

Supplementary Table S3: Enterotoxin gene primer of *Bacillus cereus*

Target gene	Primer sequence	T_m (^0C)	Product size (bp)	Reference
nheA	F = TTTCTATCGGTACTTTAAGTAATGAAATTGTA	63.5	405	Haque et al 2022
	R = AACTGTTTAATGTACTTCAACGTTTGTAAC	63.9		
cytK	F = GTAACAGATATCGGKCAAAATGCA	60.1	527	
	R = TGTTATATCCRTTAAAGAATACGTTCCA	61.3		
entFM	F = AAAGAAATTAATGGACAAACTCAAACTCA	62.0	609	
	R = GTATGTAGCTGGGCCTGTACGT	64.0		

Supplementary Table S4. Source and type of sample

Location	Type of sample (*n*=30)						Total
	Layer feed (*n*=5)	Broiler feed(*n*=5)	Cattle feed(*n*=5)	Fish feed(*n*=5)	Milk (*n*=5)	Egg (*n*=5)	
Sonapurbazar	1	1	1	1	1	1	6
Mazidee bazar	1	1	1	1	1	1	6
Gabua bazar	1	1	1	1	1	1	6
Choumohu Bazar	1	1	1	1	1	1	6
Datter hat Bazar	1	1	1	1	1	1	6
Total	5	5	5	5	5	5	30

Supplementary Table S5: Biochemical characteristics of *Bacillus cereus*

Test	Gram stain	Egg yolk reaction	Catalase	Motility	Mannitol	Citrate	Voges-Proskauer	Nitrate reduction	Indole	Starch hydrolysis	Acid from			Oxidase	Anaerobic growth
											Glucose	Lactose	Sucrose		
Result	+	+	+	+	-	+	+	+	-	+	+	-	-	-	+

Supplementary Table S6: Diagnostic outcomes of LAMP vs. PCR

Target Gene	LAMP Positive / PCR Positive (TP)	LAMP Positive / PCR Negative (FP)	LAMP Negative / PCR Positive (FN)	LAMP Negative / PCR Negative (TN)	LAMP Positive	PCR positive
nheA	18	4	1	7	22	19
cytK	11	5	0	14	16	11
entFM	21	4	1	5	25	22
Overall	49	13	2	26	63	52

(TP = true positive, FP = false positive, FN = false negative, TN = true negative)

Supplementary Table S7. Time and Cost analysis

**Parameter**	**LAMP (this study)**	**Conventional PCR**
Sample-to-result time	75-90 min	4-6 h
Cost per sample	$2.50	$5.80
Equipment cost	$150 (water bath)	$8,000 (thermal cycler)
Training required	Minimal (1 day)	Moderate (1-2 weeks)

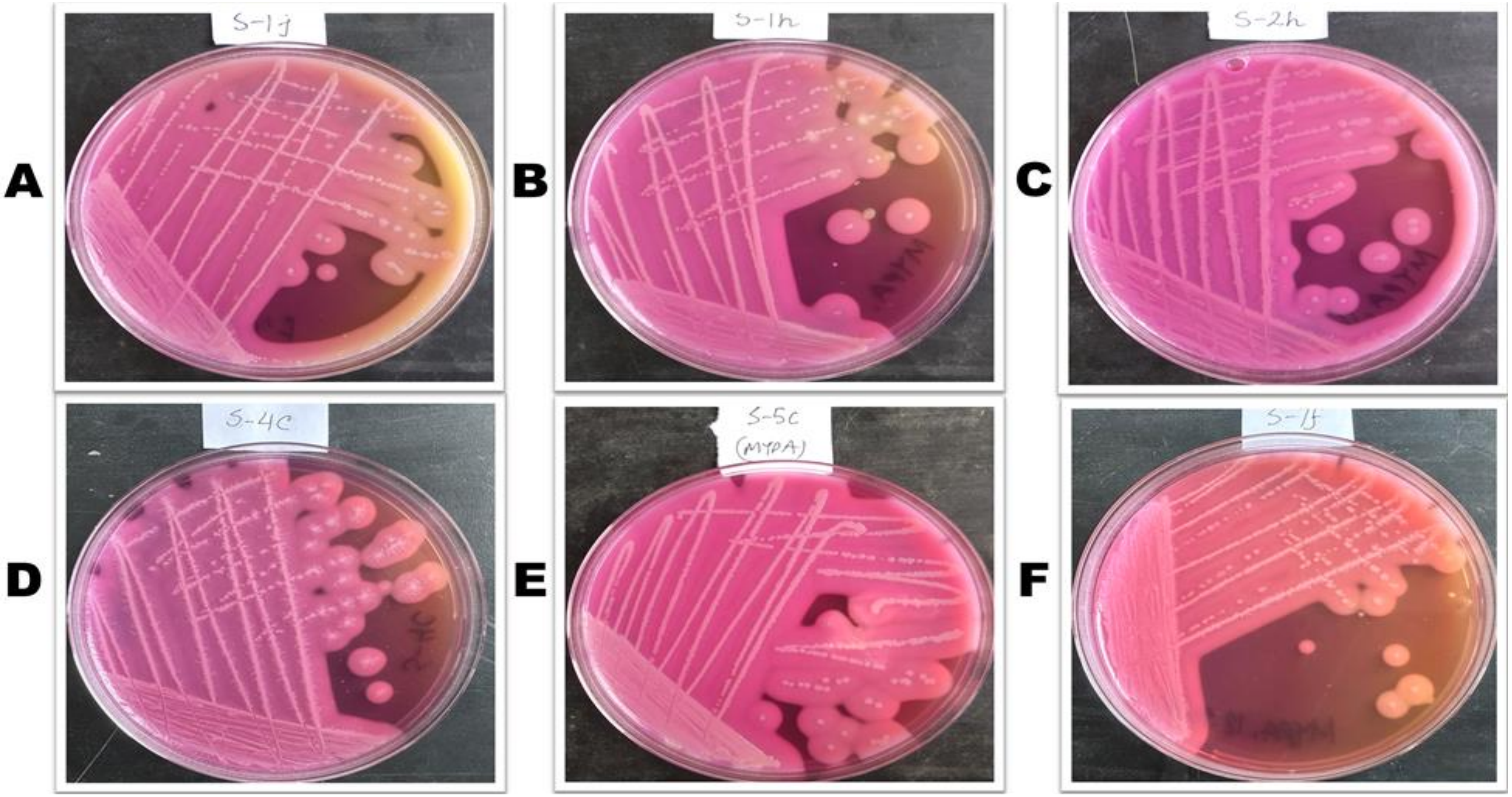

Supplementary Figure 1: Culture of isolated *Bacillus cereus* on MYPA revealed typical pink colonies with lecithinase activity; A (from layer feed), B (from Broiler feed), C (from Cattle feed), D (from fish feed), E (from milk) and F (from egg).

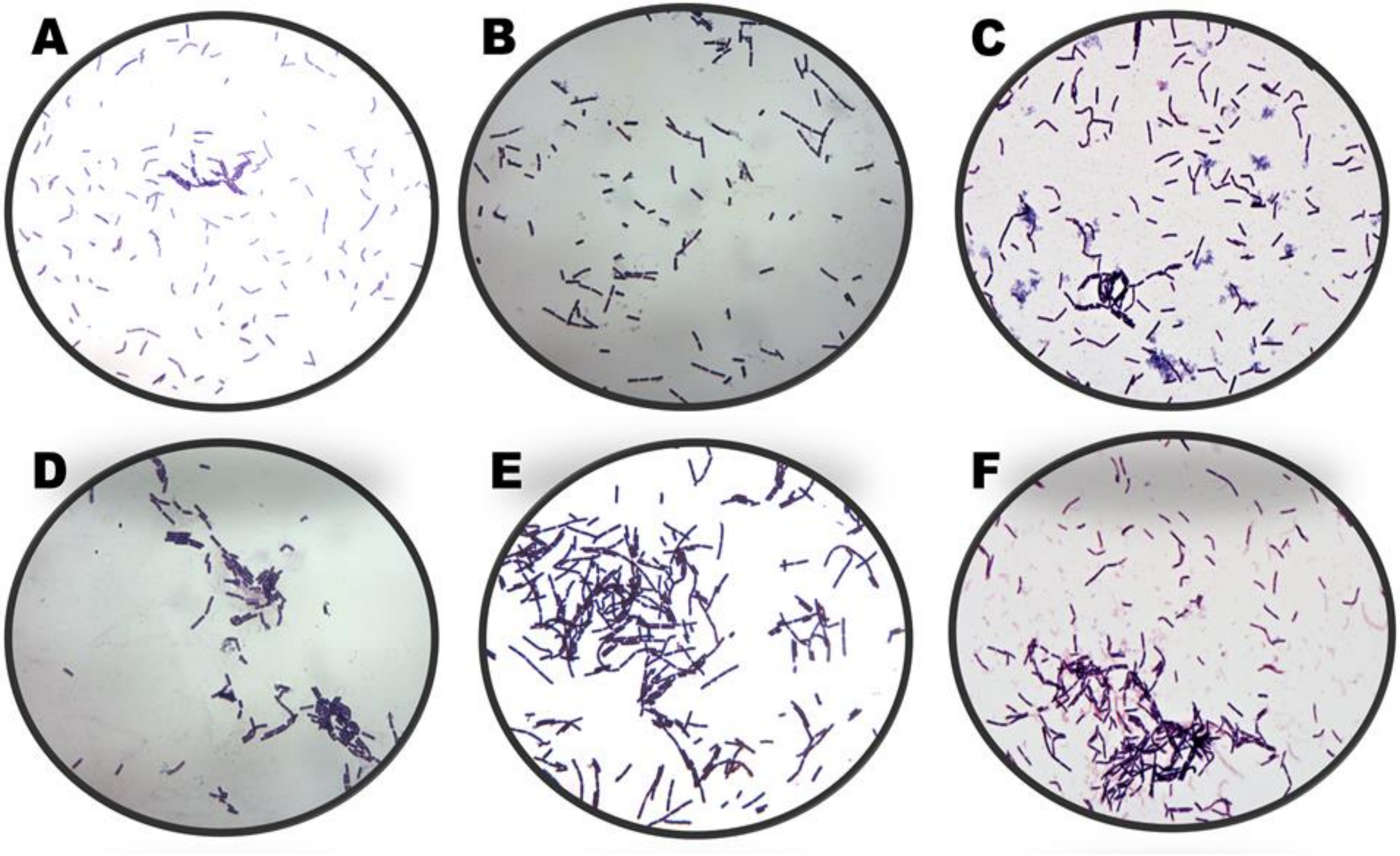

Supplementary Figure 2: Gram staining of *Bacillus cereus* showing gram positive rod shape bacteria arranged single or pair or forming chain; A (from layer feed), B (from Broiler feed), C (from Cattle feed), D (from fish feed), E (from milk) and F (from egg).
